# Microplastic and nanoplastic interactions with per- and polyfluoroalkyl substances (PFAS) in soils: a critical review of main findings and knowledge gaps

**DOI:** 10.1007/s10653-026-03419-x

**Published:** 2026-08-29

**Authors:** Pedro Augusto Soares, Guilherme Silva de Souza, Valéria Guimarães Silvestre Rodrigues, Jacqueline Zanin Lima

**Affiliations:** Department of Geotechnical Engineering, São Carlos School of Engineering, University of São Paulo, 400 Trabalhador São Carlense Ave., São Carlos, 13566-590 Brazil

**Keywords:** Bioaccumulation, Cotransport, Environmental aging, Emerging contaminants, Plastic particles, Soil contamination

## Abstract

Microplastics (MPs), nanoplastics (NPs), and per- and polyfluoroalkyl substances (PFAS) can enter soils through biosolids, compost, landfill leachate, reclaimed-water irrigation, treated textiles, and industrial releases. However, whether plastic particles modify PFAS fate in terrestrial environments remains uncertain because most evidence derives from simplified laboratory conditions. This review followed the Preferred Reporting Items for Systematic Reviews and Meta-Analyses (PRISMA) framework and identified 430 records from Scopus (*n* = 208) and Web of Science (*n* = 222). After screening, environmental-domain classification, and critical reassessment, 37 original studies were included in a two-level evidence framework comprising eight core soil or soil-relevant studies and 29 complementary mechanistic studies. PFAS–MP/NP interactions depended on polymer composition, particle size and charge, PFAS chain length and functional group, solution chemistry, weathering, organic matter, mineral coatings, and biofilms. Although pristine plastics often showed substantial PFAS adsorption in aqueous systems, their affinity frequently changed after contact with soils or other environmental matrices. Transport studies did not support a universal carrier effect. Plastic particles facilitated PFAS movement under some conditions, retarded it when particle retention dominated, and had little effect when most PFAS remained dissolved. Biological responses were also context-dependent. MPs increased PFAS bioaccumulation and reproductive toxicity in earthworms, while plant and aquatic studies showed both enhanced and reduced uptake or toxicity depending on particle properties and exposure conditions. Overall, the PFAS vector effect of MPs and NPs is conditional rather than universal. Field monitoring, intact-soil experiments, environmentally conditioned particles, realistic concentrations, complete mass balances, and long-term assessments are needed to determine when plastic-mediated processes become environmentally significant.

**Supplementary Information:**

The online version contains supplementary material available at https://doi.org/10.1007/s10653-026-03419-x.

## Introduction

Soils are major receiving compartments and long-term reservoirs for per- and polyfluoroalkyl substances (PFAS), microplastics (MPs), and nanoplastics (NPs). These contaminants can enter terrestrial environments through the application of biosolids and compost, irrigation with reclaimed water, degradation of agricultural plastics, landfill leachate, atmospheric deposition, and the release of fibers from treated textiles (Kookana et al., 2023; Prada et al., 2024; Saha et al., 2024; Schellenberger et al., 2019). Wastewater treatment and waste-management systems are particularly relevant because they can concentrate both PFAS and plastic particles in sludge and other solid residuals that may subsequently be applied to or disposed of on land (Ma et al., 2025; Prada et al., 2024). These shared pathways create multiple opportunities for PFAS and MPs/NPs to interact before and after their incorporation into soils.

Once present in the same matrix, MPs and NPs may alter PFAS partitioning by acting as sorbents, transient reservoirs, mobile carriers, or even secondary sources of fluorinated compounds. Controlled experiments indicate that these interactions depend on PFAS chain length and functional group, polymer composition, particle size, surface charge, oxidation state, and the chemistry of the surrounding solution (Bere et al., 2024; Freilinger et al., 2025; Hatinoglu et al., 2023; Llorca et al., 2018). Hydrophobic partitioning, electrostatic attraction or repulsion, hydrogen bonding, and dispersion forces may contribute simultaneously, although their relative importance varies among PFAS–polymer combinations (Simonetti et al., 2025; Wang et al., 2015). Accordingly, the environmental behavior of a short-chain perfluoroalkyl carboxylic acid associated with an oxidized polyamide particle cannot be assumed to resemble that of a long-chain sulfonate interacting with polyethylene, polypropylene, or polystyrene.

The central uncertainty is whether mechanisms identified in simplified laboratory systems remain environmentally meaningful in natural soils. Much of the available evidence was generated using pristine and monodisperse particles, aqueous solutions, short exposure periods, elevated contaminant concentrations, and homogeneous quartz-sand columns. These conditions facilitate mechanistic interpretation but do not reproduce the heterogeneity of soil organic matter, mineral surfaces, pore networks, microbial communities, moisture regimes, and competitive adsorption processes. Ateia et al. (2020) showed that real plastic materials containing additives, fillers, and irregular surfaces may behave differently from pure polymers, while Scott et al. (2021) observed substantially greater PFAS accumulation on field-incubated plastics carrying associated organic and inorganic matter than on clean particles exposed in laboratory water. Conversely, Sun et al. (2024) found that the high PFAS adsorption capacity of isolated polyamide MPs was strongly reduced after their incorporation into soil because mineral particles and biofilms covered the plastic surface. Natural organic matter may likewise enhance, inhibit, or redirect PFAS–plastic interactions through bridging, surface coating, steric effects, and competitive adsorption (Salawu et al., 2024; Tang et al., 2025).

Similar uncertainty applies to transport. Quartz-sand column studies demonstrate that PFAS and MPs can modify each other’s mobility through changes in surface charge, aggregation, competitive adsorption, and attachment to collector grains (Rong et al., 2023; Zhang et al., 2025). However, experiments using natural sediments indicate that particle size, matrix heterogeneity, mineral composition, and physical straining can weaken or decouple this cotransport behavior (Xie et al., 2025; Xu et al., 2024). Biological consequences are also context-dependent. Direct terrestrial evidence remains scarce, although Sobhani et al. (2021) reported that polyvinyl chloride (PVC) MPs increased perfluorooctane sulfonate (PFOS) and perfluorooctanoic acid (PFOA) bioaccumulation and reproductive toxicity in earthworms. Evidence from aquatic organisms and plants further indicates that MPs/NPs may either increase or attenuate PFAS bioavailability and toxicity, depending on particle size, surface chemistry, contaminant state, exposure duration, and biological endpoint (Farajizadeh et al., 2025; Gupta et al., 2024; Liu et al., 2023).

Therefore, the key scientific question is not merely whether PFAS can interact with MPs and NPs, but under which environmental conditions these interactions significantly affect PFAS retention, release, mobility, bioavailability, and toxicity in soils. Addressing this question requires a distinction between direct evidence obtained from soils and soil-relevant porous media and complementary mechanistic evidence generated in other environmental systems.

This review systematically maps the distribution of PFAS–MP/NP research across environmental domains and critically synthesizes the evidence relevant to terrestrial systems. Following the Preferred Reporting Items for Systematic Reviews and Meta-Analyses (PRISMA) framework (Page et al., 2021), the review combines direct studies conducted in soils, natural sediments, porous media, compost, biosolids, and soil organisms with selected mechanistic studies from aquatic and experimental systems. The objectives are to identify the physicochemical mechanisms governing PFAS–MP/NP interactions, evaluate how soil properties and environmental aging modify these processes, assess implications for cotransport, plant uptake, bioaccumulation, and toxicity, and determine why results obtained under idealized laboratory conditions may not be directly transferable to heterogeneous natural soils. Elucidating these coupled transport and aging mechanisms contributes to more robust predictive modeling of contaminant fate in terrestrial environments. Understanding the factors that govern these interactions in heterogeneous systems is essential not only for developing targeted soil remediation strategies and ensuring food safety, but also to provide critical insights that improve environmental risk assessments and support regulatory decisions regarding co-contaminated soil landscapes.

## Theoretical background

### Microplastics and nanoplastics: properties relevant to PFAS interactions

MPs are generally defined as synthetic polymer particles smaller than 5 mm, whereas NPs comprise the colloidal fraction, commonly placed between approximately 1 and 1000 nm, although definitions using an upper limit of 100 nm are also reported (Gigault et al., 2021; Hartmann et al., 2019). For PFAS interactions, this size distinction is relevant because decreasing particle size increases the specific surface area available for adsorption and changes particle aggregation, retention, and transport. NPs may access smaller pore spaces and remain dispersed under favorable solution conditions, but their mobility can be reduced by homoaggregation or heteroaggregation with minerals, organic matter, and other soil colloids (Bere et al., 2024; Simonetti et al., 2025).

Polymer composition determines the chemical environment encountered by PFAS at the particle surface. Polyethylene (PE), polypropylene (PP), and polystyrene (PS) are predominantly nonpolar and favor interactions associated with hydrophobic partitioning and dispersion forces. Polyamide (PA), in contrast, contains amide groups capable of participating in polar interactions and hydrogen bonding, while polyethylene terephthalate (PET) contains ester functionalities that may also affect surface polarity after weathering (Ateia et al., 2020; Salawu et al., 2024). In an investigation involving 18 polymers and eight PFAS, Freilinger et al. (2025) observed particularly strong uptake by PA materials and attributed this behavior to their amide functionalities and stronger calculated interaction energies. These results show that polymer identity cannot be represented adequately by density or buoyancy alone.

Particle morphology and material composition are also important. Environmental MPs are usually irregular fragments or fibers containing pigments, stabilizers, plasticizers, mineral fillers, and other additives, whereas many laboratory experiments employ smooth and chemically uniform beads. Ateia et al. (2020) found that real plastic materials frequently sorbed more PFAS than corresponding pure polymers, partly because of surface roughness and inorganic fillers such as talc and glass fiber. The authors also showed that natural organic matter preloaded onto the plastic surface altered contaminant uptake. Therefore, observations obtained with a single commercial polymer should not be generalized to all particles of the same nominal polymer class. Table 1 summarizes the main particle and surface characteristics that may influence PFAS adsorption, aggregation, transport, and retention in soil-relevant systems.

**Table 1:** Characteristics of representative plastic particles that may influence their interactions with PFAS

Polymer or particle characteristic	Interaction-relevant property	Potential implication for PFAS behavior
Polyethylene (PE) and polypropylene (PP)	Predominantly nonpolar surfaces	Favor hydrophobic partitioning and dispersion interactions, although additives and oxidation may alter the surface response (Ateia et al., 2020; Wang et al., 2015)
Polystyrene (PS)	Aromatic and hydrophobic structure	May promote hydrophobic and dispersion interactions; oxidation and surface functionalization can change PFAS affinity (Hatinoglu et al., 2023; Llorca et al., 2018)
Polyamide (PA)	Polar amide functional groups	Can support hydrogen bonding and stronger polar interactions, resulting in high uptake for some PFAS (Freilinger et al., 2025)
Polyethylene terephthalate (PET)	Ester-containing polymer with weathering-sensitive surface	Fragmentation and oxidation may expose additional functional groups and change electrostatic interactions (Salawu et al., 2024)
Nanoplastic size	High specific surface area and colloidal behavior	Increases available interfacial area, but mobility depends on charge, aggregation, ionic conditions, and interactions with soil colloids (Bere et al., 2024; Simonetti et al., 2025)
Irregular morphology and surface roughness	Greater heterogeneity and availability of surface domains	May increase uptake compared with smooth commercial particles and create variable adsorption behavior (Ateia et al., 2020)
Additives and mineral fillers	Modify polarity, porosity, and surface chemistry	Can create additional binding sites or alter interactions expected from the pure polymer (Ateia et al., 2020)
Weathering and oxidation	Formation of oxygenated groups and changes in roughness and charge	May enhance or suppress PFAS adsorption depending on polymer type, PFAS structure, and environmental coatings (Barhoumi et al., 2024; Ning et al., 2024)
Biofilms, organic matter, and mineral coatings	Redefine the effective particle surface	May provide new adsorption domains, compete for PFAS, or mask polymer sites in natural soils (Bhagwat et al., 2021; Scott et al., 2021; Sun et al., 2024)

Main properties of representative microplastic and nanoplastic particles that influence PFAS adsorption, aggregation, transport, and retention. The effects listed are conditional and may vary according to particle aging, PFAS structure, solution chemistry, and the surrounding environmental matrix

Environmental weathering further changes the properties that control PFAS adsorption. Ultraviolet exposure, oxidation, abrasion, and biological colonization can increase surface roughness, generate oxygen-containing functional groups, modify surface charge, and expose additives or internal adsorption domains (Barhoumi et al., 2024; Ning et al., 2024). Experiments with secondary PET MPs produced from plastic bottles showed that PFAS adsorption was sensitive to pH, ionic strength, temperature, and natural organic matter, with electrostatic repulsion increasing at higher pH and decreasing under greater ionic strength (Salawu et al., 2024). Weathering should not, however, be assumed to increase adsorption in every case. Its effect depends on the polymer, the PFAS considered, and whether newly formed surface sites remain available or become covered by environmental material.

Once present in soils, plastic surfaces are rapidly modified by mineral particles, soil organic matter, and microorganisms. Biofilms and associated extracellular polymeric substances can introduce additional binding domains and alter particle charge, hydrophobicity, and aggregation behavior (Bhagwat et al., 2021). Field-incubated plastics accumulated substantially more PFAS than clean plastics exposed only to laboratory water, indicating an important contribution from associated organic and inorganic coatings (Scott et al., 2021). In contrast, the incorporation of PA MPs into soil reduced their intrinsic PFAS adsorption capacity because soil particles and biofilms covered part of the plastic surface, although the presence of the MPs still increased the overall adsorption capacity of the soil–plastic mixture compared with soil alone (Sun et al., 2024).

These findings indicate that the interaction-relevant identity of an environmental MP or NP is not defined solely by its original polymer. Particle size, surface charge, morphology, additives, weathering history, and acquired coatings jointly determine whether the particle will retain PFAS, remain mobile, aggregate, or become immobilized in the soil matrix. Accordingly, studies based on pristine particles are useful for isolating individual mechanisms, but they represent only an initial approximation of PFAS–plastic behavior in natural soils (Ateia et al., 2020; Scott et al., 2021; Sun et al., 2024).

### PFAS: Physicochemical properties relevant to MP/NP interactions

Per- and polyfluoroalkyl substances comprise a structurally diverse group of synthetic organofluorine compounds. Their environmental persistence is largely associated with the strength of the carbon–fluorine bonds within their molecular structures. However, persistence alone does not explain their behavior in soils or their association with plastic particles. Many PFAS of environmental concern, particularly perfluoroalkyl acids, contain a fluorinated carbon chain linked to a polar or ionizable functional group. This combination results in amphiphilic properties that promote accumulation at interfaces and interactions with organic, mineral, and polymeric surfaces (Buck et al., 2011; Wang et al., 2017).

The length of the fluorocarbon chain and the terminal functional group are major controls on PFAS partitioning. At environmentally relevant pH values, perfluoroalkyl carboxylic acids and perfluoroalkyl sulfonic acids occur predominantly as negatively charged species. Their fluorinated chains contribute to hydrophobic and dispersion interactions, while the ionic headgroups control electrostatic interactions and association with polar surface sites. Increasing chain length generally increases affinity for soil organic matter, proteins, air–water interfaces, and hydrophobic polymer surfaces. Short-chain PFAS tend to remain more mobile in pore water, whereas long-chain compounds show stronger retention and greater potential for bioaccumulation (Bere et al., 2024; Kookana et al., 2023; Nguyen et al., 2020).

Compounds with different functional groups may display distinct behavior even when they have similar fluorocarbon chain lengths. Wang et al. (2015) observed that perfluorooctanesulfonamide exhibited greater partitioning onto PE, PS, and PVC than PFOS, mainly because its neutral molecular form favored hydrophobic partitioning. Similarly, Llorca et al. (2018) reported greater adsorption of sulfonates and sulfonamides than several carboxylates under the tested conditions. These findings indicate that the term “PFAS” should not be used as if all compounds had equivalent affinity for plastic or soil surfaces.

The influence of PFAS structure is inseparable from the properties of the plastic surface and the surrounding matrix. Nonpolar polymers such as PE and PP primarily favor hydrophobic and dispersion interactions, whereas polar surfaces such as PA may provide sites for hydrogen bonding and stronger electrostatic interactions (Freilinger et al., 2025; Simonetti et al., 2025). Solution pH, ionic strength, cation valence, and natural organic matter can modify these interactions by changing surface charge, electrical double-layer thickness, aggregation, and competition for adsorption sites (Hatinoglu et al., 2023; Salawu et al., 2024; Tang et al., 2025). Consequently, an adsorption trend observed for one PFAS–polymer combination should not be extrapolated to other compounds or environmental conditions. Table 2 summarizes the main physicochemical characteristics of representative PFAS groups and their expected implications for interactions with MPs/NPs and soil components.

**Table 2:** Physicochemical characteristics of representative PFAS groups relevant to their interactions with microplastics, nanoplastics, and soil components

PFAS group	Selected examples	Interaction-relevant characteristics	Expected environmental implications
Perfluoroalkyl carboxylic acids (PFCAs)	PFBA, PFHxA, PFOA	Predominantly anionic under environmental conditions. Hydrophobicity and interfacial affinity generally increase with chain length	Short-chain compounds tend to remain more mobile in pore water, whereas long-chain compounds show stronger adsorption to organic matter and hydrophobic plastic surfaces (Kookana et al., 2023; Nguyen et al., 2020)
Perfluoroalkyl sulfonic acids (PFSAs)	PFBS, PFHxS, PFOS	Anionic sulfonate headgroup and fluorinated chain. Sulfonates often show stronger interfacial affinity than carboxylates with comparable chain lengths	Greater retention may occur on hydrophobic, polar, or positively charged surfaces, particularly for long-chain compounds (Bere et al., 2024; Llorca et al., 2018)
Neutral or weakly ionizable PFAS	FOSA, fluorotelomer alcohols	Greater neutral molecular character and stronger hydrophobic partitioning than ionic PFAS. Some compounds may also be relatively volatile	May show strong partitioning to nonpolar plastics and organic phases and may act as precursors of persistent perfluoroalkyl acids (Wang et al., 2015; Washington et al., 2015)
Fluorotelomer-derived compounds	6:2 FTSA and related precursors	Intermediate transformation products containing partially fluorinated chains. Their charge and mobility depend on the terminal functional group	May be released from treated materials and subsequently transformed into more persistent terminal PFAS in soils, water, or waste systems (Schellenberger et al., 2019; Wang et al., 2017)
Ether-based PFAS alternatives	GenX, F-53B	Ether bonds and shorter fluorinated segments distinguish them from conventional PFOA and PFOS. They often show high mobility and compound-specific adsorption behavior	Their interaction with plastics, soils, and organisms cannot be predicted directly from legacy PFAS, and substitution does not necessarily indicate lower environmental risk (Chen et al., 2024; Kwiatkowski et al., 2020)

PFBA: perfluorobutanoic acid; PFHxA: perfluorohexanoic acid; PFOA: perfluorooctanoic acid; PFBS: perfluorobutane sulfonate; PFHxS: perfluorohexane sulfonate; PFOS: perfluorooctane sulfonate; FOSA: perfluorooctane sulfonamide; FTSA: fluorotelomer sulfonic acid; GenX: hexafluoropropylene oxide dimer acid; F-53B: 6:2 chlorinated polyfluorinated ether sulfonate

Once released into soils, PFAS partition among pore water, soil organic matter, mineral surfaces, air–water interfaces, organisms, and plastic particles. MPs and NPs therefore represent one potential partitioning phase within a considerably more complex system. Their importance depends on whether PFAS affinity for plastic surfaces is sufficient to compete with adsorption to soil organic matter and minerals. It also depends on whether the particles remain mobile or are retained by aggregation, straining, biofilm formation, or mineral coatings (Kookana et al., 2023; Sun et al., 2024).

PFAS precursors and polymer-associated fluorinated compounds introduce an additional source of uncertainty. Side-chain fluorinated polymers and other treated materials may release PFAS progressively through abrasion, washing, oxidation, and environmental transformation (Schellenberger et al., 2019; Washington et al., 2015; Zhao et al., 2025b, 2025c). Plastic particles may consequently act not only as surfaces that adsorb PFAS from the environment, but also as sources that release fluorinated substances already present in the original material. This distinction is important when interpreting PFAS concentrations associated with environmental MPs/NPs.

### PFAS interactions with microplastics and nanoplastics in soil systems

Interactions between PFAS and MPs/NPs should not be understood as a single and permanent association. Instead, PFAS can partition dynamically among pore water, plastic surfaces, soil organic matter, minerals, air–water interfaces, and biological tissues. Plastic particles may consequently function as sorbents, temporary reservoirs, mobile carriers, or sources of previously incorporated fluorinated compounds. Their net influence depends on the strength and reversibility of PFAS adsorption, the mobility of the particles, and the capacity of the surrounding soil matrix to compete for the same compounds (Kookana et al., 2023; Salawu et al., 2024; Sun et al., 2024).

#### Co-occurrence and shared contamination pathways

The co-occurrence of PFAS and MPs/NPs in terrestrial environments is favored by common sources and transport pathways. Wastewater treatment, landfill leachate, biosolids, compost, treated textiles, industrial waste, and urban runoff may simultaneously introduce plastic particles and fluorinated compounds into soils or soil amendments (Ma et al., 2025; Prada et al., 2024; Saha et al., 2024; Schellenberger et al., 2019). Textiles are a relevant example because washing and weathering may release both synthetic fibers and compounds derived from side-chain fluorinated polymers. These fluorinated coatings and their precursors may subsequently form mobile perfluoroalkyl acids (Schellenberger et al., 2019; Zhao et al., 2025b). Likewise, biosolids can concentrate contaminants removed from wastewater and transfer them to agricultural soils during land application.

Field observations confirm that both contaminant groups may occur in the same waste-management systems. Prada et al. (2024), for example, detected MPs and PFAS throughout linked landfill and wastewater treatment systems and found that both were transferred preferentially to solid residuals, particularly biosolids. Saha et al. (2024) also detected PFAS associated with plastic materials in yard-waste compost. These findings demonstrate overlapping exposure pathways, although co-occurrence alone does not establish that PFAS are adsorbed onto MPs or transported by them. Evidence of interaction requires measurements of particle-associated PFAS, adsorption or desorption, changes in mobility, or altered biological uptake. Field evidence from Taihu Lake further showed that MP morphology and biofilm development were associated with the distribution and sediment accumulation of selected PFAS, including HFPO-DA (Liu et al., 2024).

Plastic materials can also represent primary or secondary PFAS sources. Side-chain fluorinated polymers, water-repellent coatings, and multilayer textiles may release terminal PFAS or their precursors through abrasion, washing, oxidation, and environmental transformation (Schellenberger et al., 2019; Washington et al., 2015; Zhao et al., 2025b, 2025c). Therefore, PFAS detected on environmental plastic particles may result either from adsorption after environmental release or from fluorinated substances originally incorporated into the material. Distinguishing between these pathways is necessary to determine whether MPs/NPs are functioning as external sorbents or contaminant sources.

#### Adsorption and desorption mechanisms

PFAS adsorption onto plastic particles is governed by several non-covalent interactions that may occur simultaneously. Hydrophobic partitioning and dispersion forces promote contact between fluorinated chains and nonpolar polymer domains, whereas electrostatic attraction or repulsion depends on the charge of the PFAS headgroup and the effective surface charge of the particle. Hydrogen bonding and other polar interactions become more relevant when polymers contain amide, ester, hydroxyl, carbonyl, or carboxyl groups, either in their original structure or after environmental oxidation (Freilinger et al., 2025; Hatinoglu et al., 2023; Simonetti et al., 2025; Wang et al., 2015).

The contribution of each mechanism varies among polymer–PFAS combinations. Freilinger et al. (2025) found substantially stronger interactions between PFAS and polyamide than between the same compounds and polyethylene, reflecting the contribution of amide functional groups. In contrast, PFAS adsorption onto polyethylene, polypropylene, and polystyrene is generally associated with nonpolar interactions, surface roughness, and polymer free volume. Computational and experimental results for PFOS and polypropylene NPs indicated that dispersion forces contributed strongly to complex formation, while PFOS could associate with both external and internal domains of the particle (Simonetti et al., 2025).

PFAS molecular structure also controls adsorption. Increasing fluorocarbon chain length generally strengthens partitioning onto hydrophobic surfaces, although the terminal functional group and ionization state may modify this tendency. Sulfonates and sulfonamides have shown greater affinity than several carboxylates under comparable experimental conditions, while short-chain PFCAs commonly remain more strongly associated with the aqueous phase (Bere et al., 2024; Llorca et al., 2018; Wang et al., 2015). Consequently, adsorption results obtained with PFOA or PFOS cannot be directly extended to all legacy and emerging PFAS.

Adsorption should also be distinguished from irreversible immobilization. PFAS retained on the surface or within the polymer may desorb when solution chemistry, contaminant concentration, or competitive conditions change. Adsorbed compounds may therefore return to pore water during dilution, changes in pH or ionic composition, particle degradation, or passage through biological systems. The environmental significance of a plastic particle depends not only on the quantity initially adsorbed, but also on whether the association persists during transport and whether desorption occurs at locations where exposure is possible.

#### Influence of soil properties, solution chemistry, weathering, and biofilms

The chemistry of pore water affects both PFAS adsorption and particle stability. At increasing pH, plastic and mineral surfaces often become more negatively charged, which can increase electrostatic repulsion against anionic PFAS and reduce adsorption. Higher ionic strength compresses the electrical double layer and may reduce repulsion between similarly charged surfaces. Divalent cations can further promote association through charge screening or bridging, although the magnitude and direction of these effects depend on the polymer, PFAS structure, and mineral composition (Hatinoglu et al., 2023; Salawu et al., 2024; Zhang et al., 2025).

Natural organic matter has no uniform effect on PFAS–plastic interactions. Dissolved organic molecules may compete with PFAS for surface sites, form coatings that reduce direct contact with the polymer, or stabilize particles through steric repulsion. Under other conditions, adsorbed organic matter provides additional hydrophobic or polar domains capable of retaining PFAS. Salawu et al. (2024) observed reduced PFAS adsorption onto secondary PET MPs in the presence of natural organic matter, whereas Scott et al. (2021) found considerably greater PFAS accumulation on field-incubated plastics covered by organic and inorganic material than on clean particles exposed in laboratory water. These contrasting results show that the effect of organic matter depends on its composition, concentration, state of association with the particle, and interaction with other environmental constituents.

Weathering can modify PFAS adsorption by increasing surface roughness, producing cracks and pores, altering crystallinity, and generating oxygen-containing functional groups. Naturally aged PS particles showed greater PFAS adsorption than virgin particles, which was attributed to oxidation, morphological changes, and biofilm development (Barhoumi et al., 2024). Laboratory-aged PE, PET, and PS particles have also shown altered PFOA affinity following ultraviolet or chemical oxidation, although the resulting effect varied among polymers and aging procedures (Ning et al., 2024). Weathering therefore changes the available adsorption mechanisms but does not necessarily increase PFAS retention under every environmental condition. Biodegradation and surface-water aging of PLA likewise increased PFOA adsorption as oxidation and hydrolysis generated hydroxyl, carbonyl, and carboxyl groups on the polymer surface (Tumrani et al., 2025).

Biofilms further modify the effective surface of MPs/NPs. Extracellular polymeric substances contain proteins, polysaccharides, lipids, and charged functional groups that may create new PFAS-binding domains and alter aggregation or surface charge (Bhagwat et al., 2021; Song et al., 2025). Field-incubated plastics and laboratory particles with developed biofilms frequently show greater contaminant adsorption than pristine materials. In soils, however, biological coatings occur together with mineral adhesion and organic matter deposition. Sun et al. (2024) found that isolated PA MPs had high PFAS adsorption capacity, but this capacity decreased substantially after the particles were mixed with soil because their surfaces became covered by soil particles and biofilm-like material. Even so, the soil–MP mixture retained more PFAS than soil alone.

**Fig. 1:**
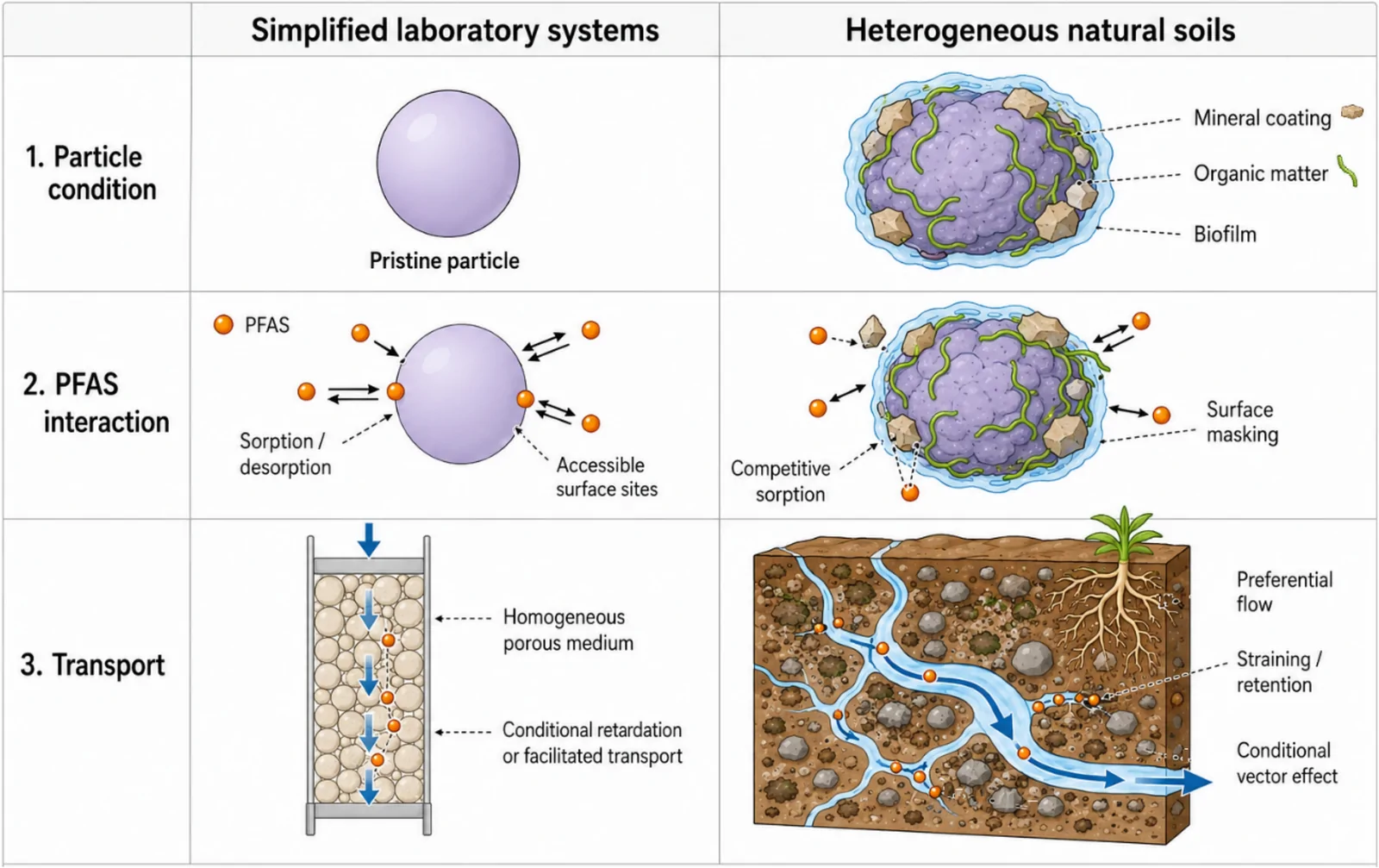
Conceptual comparison of PFAS–MP/NP interactions in simplified laboratory systems and heterogeneous natural soils

These findings indicate that aging and environmental conditioning can produce opposite outcomes. Surface oxidation and biofilm development may create additional adsorption domains, while mineral coatings and competitive soil adsorption may conceal those same domains. The behavior of environmentally conditioned particles therefore cannot be inferred only from comparisons between pristine and artificially aged plastics. Figure 1 presents the main processes that can enhance or suppress PFAS–MP/NP interactions under simplified laboratory conditions and in heterogeneous natural soils.

Laboratory experiments commonly employ pristine particles, homogeneous quartz sand, controlled solution chemistry, elevated contaminant concentrations, and short exposure periods, conditions that favor the observation of adsorption and cotransport mechanisms. In natural soils, aging, mineral coatings, organic matter, biofilms, competitive adsorption, physical straining, preferential flow, and temporal variability may enhance, suppress, or reverse these interactions.

#### Cotransport, retention, and mobility in soil matrices

Cotransport occurs only when PFAS remain associated with particles that are themselves mobile. Strong adsorption onto an immobilized MP may retard PFAS migration, whereas adsorption onto a stable and mobile NP may increase transport relative to PFAS movement in the dissolved phase. Particle attachment, aggregation, pore-throat straining, and deposition on mineral surfaces therefore determine whether plastics behave as carriers or retention phases.

Column experiments with quartz sand show that the presence of PFOA can alter MP surface charge and mobility, while MPs may reduce or enhance PFOA transport depending on particle charge and solution chemistry. Rong et al. (2023) found that PFOA inhibited the transport of negatively charged MPs but increased the mobility of positively charged particles. Despite these contrasting effects on the particles, both MP types reduced PFOA transport because the MPs were less mobile than dissolved PFOA. Zhang et al. (2025) similarly found that PS-MPs generally retarded PFOA through adsorption, but competitive adsorption at pH 5 promoted PFOA migration under specific conditions.

Particle size adds another level of control. In natural sediment columns, smaller PS particles moved more readily than larger particles, while larger MPs were retained by attachment and physical straining (Xu et al., 2024). PFOA increased MP mobility under some acidic conditions by making particle surfaces more negatively charged, but this effect became weak at a higher pH because the sediment buffered the influent chemistry. Xie et al. (2025) observed that highly mobile PS-NPs produced little change in PFOA transport, whereas larger and less mobile MPs caused a small increase in PFOA retention. The effect of plastics on PFAS movement therefore depends on the balance between PFAS affinity for the particle and the transport behavior of the particle within the porous matrix.

These studies demonstrate that the expression “MPs act as PFAS vectors” is insufficient without specifying the environmental conditions. The same plastic particle may facilitate PFAS movement in one system and retard it in another. Quartz-sand columns are useful for identifying electrostatic and hydrodynamic mechanisms, but natural soils introduce variable mineralogy, organic matter, aggregation, preferential flow, air–water interfaces, and unsaturated conditions that may weaken or reverse the patterns observed in homogeneous media.

#### Plant uptake, bioaccumulation, trophic transfer, and toxicity

PFAS–plastic interactions may modify biological exposure by changing the freely dissolved PFAS fraction, particle ingestion, desorption within organisms, or the ability of NPs to cross biological barriers. Depending on the exposure system, adsorption can either reduce PFAS bioavailability by lowering the dissolved concentration or increase uptake by delivering PFAS together with ingested or internalized particles.

Direct evidence from soil organisms remains limited. Sobhani et al. (2021) reported that PVC MPs increased the bioaccumulation factors of PFOA and PFOS in earthworms and reduced reproductive performance under combined exposure. This study supports the possibility that ingestion of contaminated MPs can enhance PFAS transfer to terrestrial invertebrates. However, the use of pristine PVC particles and relatively high contaminant concentrations limits extrapolation to field soils containing aged particles and competing adsorption phases.

Plant studies have produced contrasting responses. In soybean sprouts, adsorption of PFOS onto PS MPs and NPs reduced the freely available PFOS fraction and attenuated several acute phytotoxic effects. At the same time, the presence of PFOS increased the internalization of PS-NPs by modifying particle surface properties (Liu et al., 2023). Experiments with Eichhornia crassipes also showed that PS-MPs modified the accumulation and translocation of PFOA, PFOS, GenX, and F-53B differently, indicating that the outcome depends on PFAS structure and plant uptake pathways (Chen et al., 2024). These studies were conducted in aquatic or hydroponic systems, and equivalent evidence involving crops grown in contaminated soils remains scarce.

Aquatic toxicological studies provide supporting mechanistic evidence but should not be treated as direct substitutes for terrestrial experiments. PS-MPs enhanced PFOS toxicity during zebrafish development, producing oxidative stress, developmental effects, and altered neurobehavioral responses (Gupta et al., 2024). PS-NPs also increased PFOA uptake and oxidative stress in Pacific oysters, with stronger effects for smaller particles (Farajizadeh et al., 2025). In adult zebrafish, natural organic matter intensified the combined effects of NPs and F-53B by altering tissue responses, gut microbial communities, and gene expression (Junaid et al., 2024).

Evidence for trophic transfer specifically mediated by PFAS-bearing MPs/NPs in terrestrial food webs is still insufficient. Uptake by soil invertebrates and plants establishes possible entry points, but transfer through predators and higher trophic levels has not been adequately demonstrated under realistic soil conditions. Current evidence therefore supports a potential exposure pathway rather than a quantified contribution to trophic magnification.

Overall, the biological effect of PFAS–plastic association cannot be classified consistently as synergistic or antagonistic. Increased particle-mediated uptake may intensify toxicity, whereas strong adsorption may reduce the freely dissolved PFAS fraction and temporarily lower exposure. The direction of the response depends on desorption kinetics, particle size, polymer type, PFAS structure, exposure route, organism, and environmental matrix. These variables must be considered together when assessing combined risk in soils.

## Literature review methodology

### Search strategy and record management

The literature search and screening procedures were structured according to the PRISMA protocol (Preferred Reporting Items for Systematic Reviews and Meta-Analyses) (Page et al., 2021) to ensure transparency, replicability, and methodological rigor. Scopus and Web of Science were selected because of their broad coverage of peer-reviewed environmental and interdisciplinary research. No initial restriction on publication year was applied. The final search was conducted on November 12, 2025, and the retrieved publications covered the period from 2014 to 2025.

Several combinations of terms were tested during an exploratory stage to identify a search expression capable of retrieving studies addressing both PFAS and plastic particles. The conceptual strategy was adapted to the syntax of each database. The final expressions were:

TITLE-ABS-KEY (“PFAS” OR “per- and polyfluoroalkyl” OR “perfluoroalkyl” OR “polyfluoroalkyl” OR “perfluorinated” OR “polyfluorinated” OR “PFOA” OR “perfluorooctanoic acid” OR “PFOS” OR “perfluorooctane sulfonate” OR “forever chemicals”) AND (“microplastic” OR “microplastics” OR “nanoplastic” OR “nanoplastics”).

**Fig. 2:**
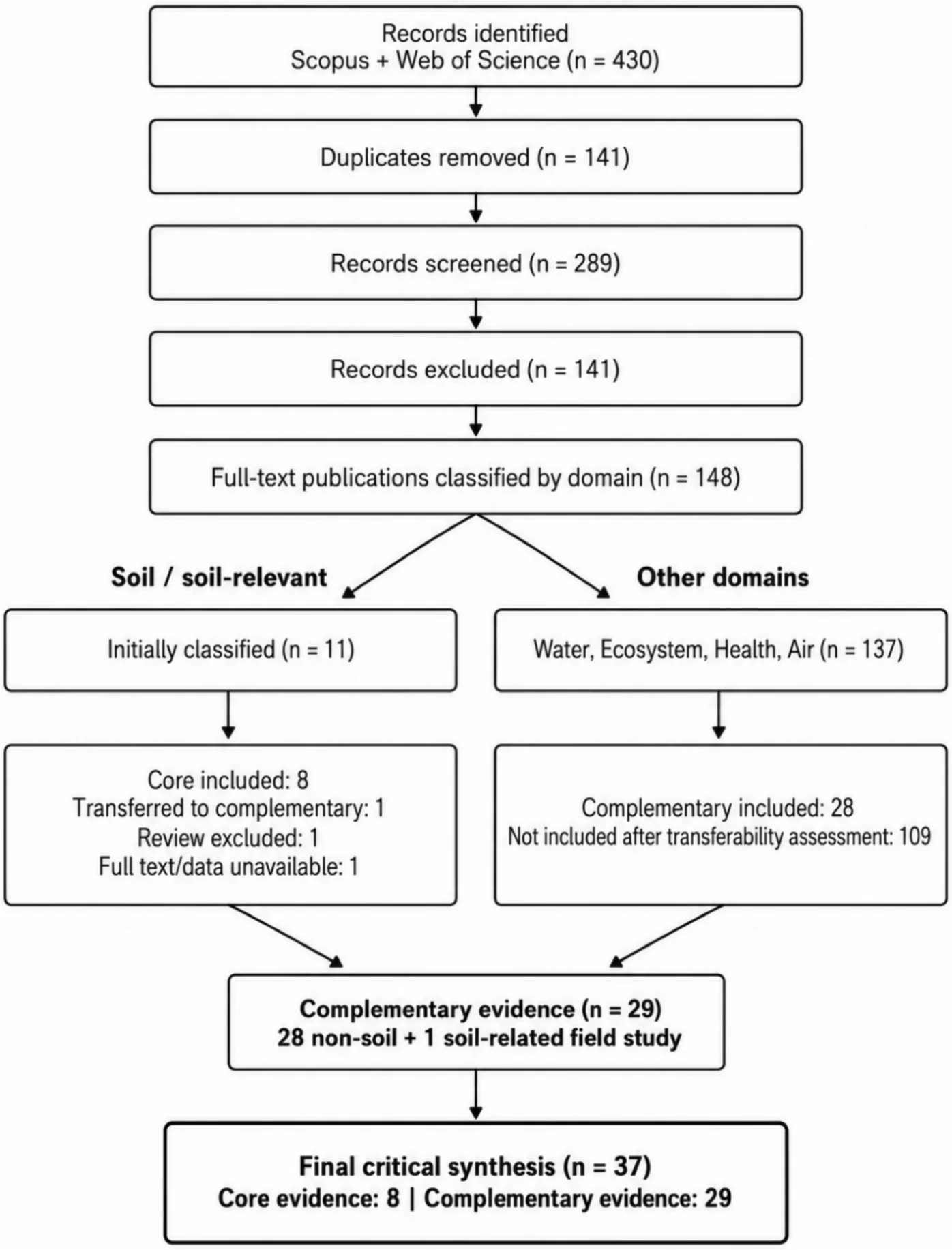
Literature identification, screening, environmental-domain classification, and two-level evidence-selection procedure

The search identified 430 records across the two databases: Scopus (*n* = 208) and Web of Science (*n* = 222). Records were exported in spreadsheet format and merged into a single dataset. After the removal of 141 duplicates, 289 unique publications remained for title and abstract screening. Review articles, conference papers, non-peer-reviewed documents, and studies written in languages other than English were not included in the primary evidence synthesis. The complete identification, screening, classification, and evidence-selection procedure is summarized in Fig. 2.

### Screening and environmental-domain classification

During title and abstract screening, 141 records were excluded because they did not address the joint occurrence, interaction, combined exposure, or environmental relationship between PFAS and MPs/NPs, or because they did not provide empirical evidence relevant to the review objectives. The remaining 148 publications underwent full-text assessment and were classified according to their principal environmental context (Fig. 2).

For descriptive mapping, the studies were assigned to five domains: Soil, Water, Ecosystem, Health, and Air (Table SM1). Eleven publications were initially classified as Soil or soil-relevant, while 137 were distributed among the other four domains (Fig. 2). The classification was used to characterize the distribution of the literature and identify the limited representation of terrestrial research. It was not subsequently treated as an automatic exclusion criterion, because studies conducted in aquatic, biological, or engineered systems could provide mechanisms relevant to PFAS–MP/NP behavior in soils.

The domain classification supported the bibliometric mapping but did not determine inclusion in the critical synthesis. Eligibility for the synthesis was assessed separately through the two-level evidence framework described below.

### Two-level evidence framework

The critical synthesis was organized using a two-level evidence framework. The first level comprised core soil and soil-relevant evidence. Studies were included in this group when they investigated particle-associated PFAS occurrence, adsorption, transport, retention, biological uptake, or combined toxicity in soils, compost, terrestrial organisms, natural sediment, or porous media directly relevant to contaminant migration in terrestrial systems.

Of the 11 publications initially classified as Soil or soil-relevant, eight original studies met the criteria for inclusion in the core evidence set (Fig. 2). One original field study reported the co-occurrence and mass distribution of PFAS and MPs in landfill–wastewater treatment systems but did not directly test adsorption, cotransport, or another causal interaction. This study was retained as complementary contextual evidence rather than as core evidence. One publication was excluded from the primary evidence set because it was a review article, and one was excluded because the full text and the methodological information required for data extraction could not be obtained.

The second level comprised complementary original evidence. The 137 publications assigned to the Water, Ecosystem, Health, and Air domains were reassessed to determine whether their findings could inform PFAS–MP/NP processes in soils (Fig. 2). Studies were retained when they provided evidence relevant to at least one of the following processes:

1. PFAS adsorption or desorption on MPs/NPs;
2. Effects of polymer composition, particle size, morphology, or surface charge;
3. Effects of weathering, oxidation, biofilms, or environmental coatings;
4. Effects of pH, ionic strength, cation type, or natural organic matter;
5. Cotransport, aggregation, attachment, or retention in porous media;
6. Changes in plant or organism uptake, bioaccumulation, or toxicity;
7. Field co-occurrence capable of informing shared sources or environmental exposure pathways.

This reassessment resulted in the inclusion of 28 original studies from the non-soil domains. Together with the field co-occurrence study retained from the soil-related group, the complementary evidence set contained 29 original articles. The original domain, PFAS, and main mechanism or process of the complementary studies are summarized in Table SM2. The remaining 109 non-soil publications were not included in the critical synthesis because they did not provide sufficiently transferable evidence for the defined mechanistic or exposure categories. Review articles were used only to support terminology, contextual interpretation, and reference tracking and were not counted as primary evidence.

The final critical synthesis therefore comprised 37 unique original research articles: eight core soil or soil-relevant studies and 29 complementary studies (Fig. 2). The complementary literature was used to interpret mechanisms and evaluate transferability, whereas conclusions about terrestrial behavior were based primarily on the core evidence.

### Data extraction and critical synthesis

For each study, information was extracted on environmental matrix, PFAS compound, polymer type, particle size, particle condition, experimental concentration, exposure duration, solution or soil properties, analytical method, interaction mechanism, transport behavior, biological endpoint, and principal limitation.

The core and complementary studies were not treated as equivalent forms of evidence. Results obtained directly in soils or soil-relevant matrices were given greater weight when drawing conclusions about terrestrial behavior. Evidence from aquatic, hydroponic, or simplified experimental systems was used to explain mechanisms, identify controlling variables, and evaluate whether observed effects could reasonably occur in soils.

The synthesis was organized by process rather than by individual article. Studies were compared according to adsorption and desorption, environmental conditioning, cotransport and retention, biological uptake, toxicity, and methodological realism. Particular attention was given to differences between pristine and aged particles, homogeneous and heterogeneous matrices, short-term and long-term experiments, and nominal adsorption capacity and environmentally relevant particle-mediated transport.

## Bibliometric mapping of research trends and evidence gaps

**Fig. 3:**
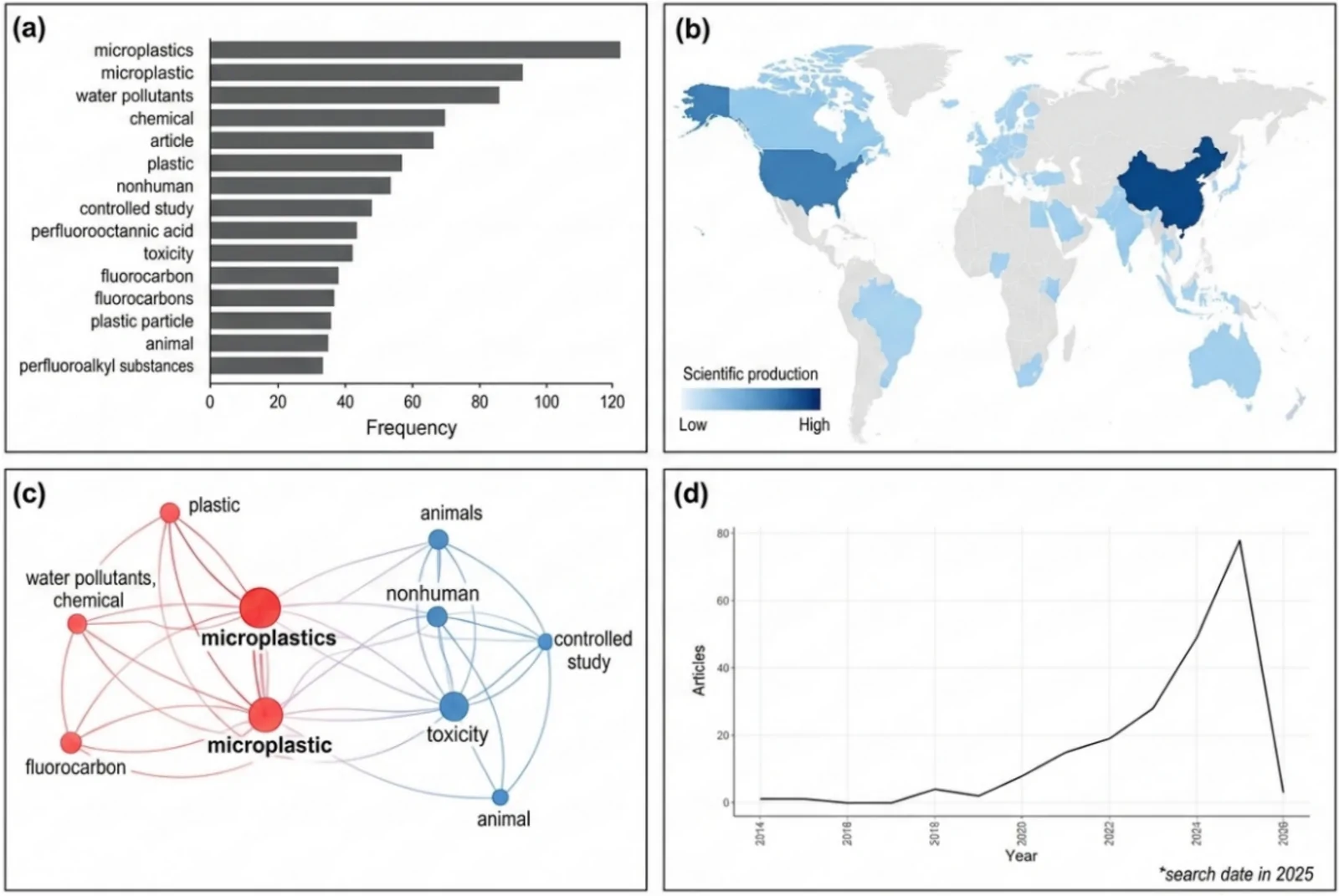
Bibliometric characteristics of research on PFAS–MP/NP interactions: (a) frequency of the 15 most recurrent indexed terms; (b) scientific production by country; (c) keyword co-occurrence network showing the principal thematic clusters; and (d) annual scientific production from 2014 to the search date in 2025. In panel (c), node size represents term frequency, line thickness represents co-occurrence strength, and colors distinguish thematic clusters

The indexed-term frequency analysis was performed using the indexed metadata of the complete bibliometric dataset rather than only the studies included in the final critical synthesis. Consequently, the resulting profile reflects the overall thematic structure of the published literature on PFAS–MP/NP interactions. Terms associated with microplastics, aquatic contamination, toxicity, chemical exposure, and experimental organisms occurred more frequently than terms specifically related to soils, vadose-zone transport, mineral surfaces, or terrestrial biota. The prominence of “microplastic” and “microplastics” indicates that the broader literature remains centered on the plastic component, whereas the behavior of individual PFAS classes and the influence of soil properties receive comparatively less attention. Because the metadata also contained database indexing terms such as “article”, “nonhuman”, and “controlled study”, the panel in Fig. 3a should be interpreted as an indexed-term profile of the complete bibliometric corpus rather than as an author-keyword analysis or a representation of the final set of selected studies.

The keyword co-occurrence network separated the literature into two closely connected research orientations (Fig. 3c). One cluster was dominated by terms related to particles, environmental occurrence, water pollutants, adsorption, and contaminant transport. The second was associated with toxicity, oxidative stress, animals, and controlled exposure experiments. The connection between these clusters reflects the prevailing interpretation of MPs/NPs as both environmental particles and possible carriers of chemical contaminants. However, the limited occurrence of soil-specific terms indicates that the connection between physicochemical interactions and terrestrial exposure remains underdeveloped.

Geographically, scientific production was concentrated in a relatively small number of countries, with China and the United States contributing the largest shares of publications (Fig. 3b). This concentration may influence the environmental matrices, contaminant profiles, and experimental approaches represented in the literature. Regions with extensive agricultural reuse of biosolids, poorly controlled waste disposal, or different soil and climatic conditions remain less represented, limiting the geographical transferability of current findings.

Annual scientific production increased markedly after 2020 and continued to rise through the end of the search period (Fig. 3d). This growth confirms that PFAS–MP/NP interactions constitute a recent and rapidly developing research field. Nevertheless, the increase in publication volume has not been accompanied by a comparable expansion of long-term soil studies, field monitoring, or experiments using environmentally conditioned particles. The keyword structure, geographical distribution, and temporal development of the literature are presented in Fig. 3.

**Fig. 4:**
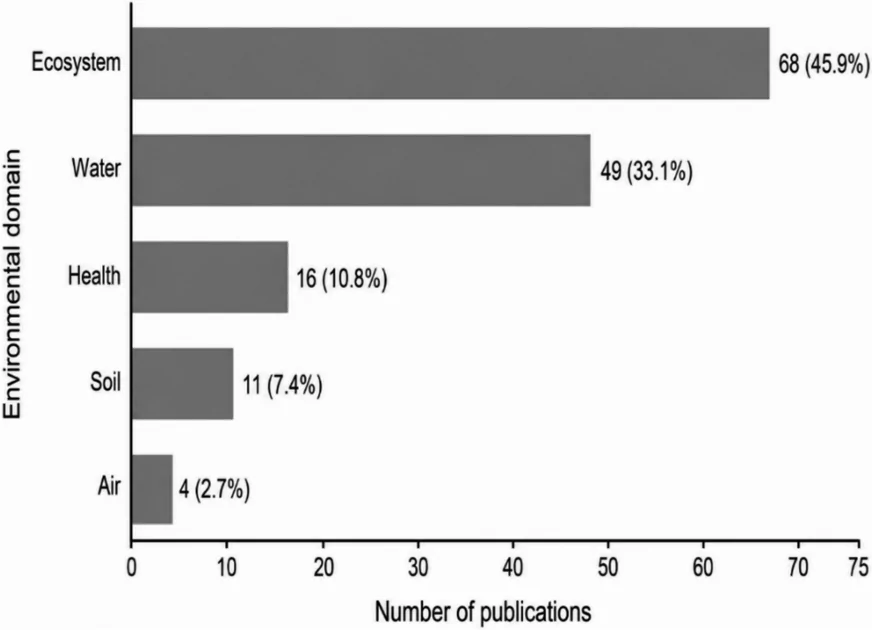
Distribution of the 148 screened publications among the Ecosystem, Water, Health, Soil, and Air environmental domains

The environmental-domain classification revealed a marked imbalance in the available literature. Studies classified under the Ecosystem and Water domains represented 45.9 and 33.1% of the screened publications, respectively, whereas Health accounted for 10.8%. Soil-related research represented 7.4% of the dataset, and studies focused on atmospheric processes accounted for 2.7%. Thus, most available evidence on PFAS–MP/NP interactions has been generated in aquatic systems, biological exposure models, or broader ecosystem contexts rather than in terrestrial matrices. Figure 4 shows the distribution of the screened publications across the five environmental domains.

The bibliometric results therefore provide more than a description of publication patterns. They identify a structural separation between the dominant aquatic and ecotoxicological literature and the smaller body of evidence available for soils. This imbalance supports the two-level evidence framework adopted in this review: direct terrestrial studies provide the primary basis for conclusions about soils, whereas studies from other environmental domains are used to explain mechanisms and assess their possible transferability to heterogeneous terrestrial systems.

## Critical synthesis of PFAS–MP/NP interactions in soil-relevant systems

### Profile and limitations of the core evidence

The direct evidence available for terrestrial and soil-relevant systems remains limited in both quantity and experimental diversity. Among the eight core studies identified in this review, five used saturated column experiments, one used batch adsorption tests in soil, one evaluated bioaccumulation and reproductive effects in earthworms, and one investigated the occurrence and distribution of PFAS associated with plastics in compost. Thus, the evidence base is dominated by short-term transport experiments rather than by long-term observations of contaminant behavior in intact soils.

Polystyrene and PFOA were the most frequently investigated plastic and PFAS, respectively. Other combinations included PTFE with PFOA and PFPeA, polyamide with four legacy and emerging PFAS, and PVC with PFOA and PFOS. This narrow selection does not represent the diversity of polymers, particle morphologies, additives, surface conditions, and PFAS structures occurring in terrestrial environments. The predominance of polystyrene appears to reflect the availability of standardized particles for controlled experiments rather than evidence that this polymer is the principal carrier of PFAS in soils.

The experimental matrices were also restricted. Quartz sand was frequently used as a model porous medium because its mineralogical uniformity facilitates the interpretation of electrostatic and hydrodynamic processes. However, quartz columns do not reproduce the organic matter, clay minerals, metal oxides, aggregates, preferential flow paths, and microbial coatings found in natural soils. Studies using natural sediment provided greater matrix realism, but the sediment was generally homogenized, amended with quartz sand, or evaluated under saturated and short-term conditions. Consequently, even the more environmentally representative column experiments did not reproduce the structural and temporal variability of field soils. The experimental characteristics, principal contributions, and main limitations of the eight core studies are summarized in Table 3.

**Table 3:** Experimental characteristics, principal findings, and limitations of the core studies addressing PFAS–MP/NP interactions in soil or soil-relevant systems

Study and matrix	Plastic and PFAS	Experimental approach	Principal contribution	Main limitation
Rong et al. (2023), quartz sand	Charged PS-MPs and PFOA	Saturated column experiments under different PFOA concentrations and ionic strengths	PFOA reduced the transport of negatively charged MPs and increased the transport of positively charged MPs. Both particle types reduced PFOA mobility because the MPs were less mobile than dissolved PFOA	Pristine and uniformly charged particles, homogeneous quartz sand, elevated concentrations, and saturated short-term conditions
Zhang et al. (2025), quartz sand	PS-MPs and PFOA	Saturated column experiments varying pH, ionic strength, and cation type	PS-MPs generally retarded PFOA transport through adsorption and particle deposition. At pH 5, competitive adsorption on quartz surfaces increased PFOA mobility	Single polymer and PFAS, simplified mineral medium, no organic matter, and no particle aging
Zhao et al. (2025a), porous medium	PTFE-MPs with PFOA and PFPeA	Column experiments varying pH, ionic strength, ion valence, PFAS concentration, and chain length	Long-chain PFOA produced a stronger increase in PTFE mobility than short-chain PFPeA, indicating that PFAS structure can modify particle aggregation and transport	Only one fluoropolymer and two PFAS were considered under saturated laboratory conditions
Xie et al. (2025), natural sediment amended with quartz sand	PS-NPs, PS-MPs, and PFOA	Saturated column experiments comparing particle sizes and pH conditions	Highly mobile NPs produced little change in PFOA transport, whereas larger and less mobile MPs slightly increased PFOA retention	One sediment, one polymer, pristine particles, short exposure, and limited representation of natural soil structure
Xu et al. (2024), natural sediment mixed with quartz sand	PS particles of different sizes and PFOA	Packed-column experiments evaluating particle size, PFOA presence, and influent pH	Small particles showed high breakthrough, whereas larger MPs were retained by attachment and physical straining. PFOA altered MP mobility under selected conditions	Homogenized sediment, low organic matter, pristine spherical particles, and PFOA concentrations above typical environmental levels
Sun et al. (2024), paddy soil	PA-MPs with PFOA, 6:2 FTSA, PFHxS, and GenX	Batch adsorption experiments comparing MP, soil, and MP–soil mixtures	Isolated PA-MPs had much higher PFAS adsorption than soil. Soil particles and biofilm-like coatings strongly reduced MP adsorption, although the MP–soil mixture retained more PFAS than soil alone	One soil and polymer, high experimental PFAS concentrations, short conditioning periods, and no weathered particles
Sobhani et al. (2021), soil and earthworms	PVC-MPs with PFOA and PFOS	Soil exposure followed by bioaccumulation and reproduction assays	PVC-MPs increased PFAS bioaccumulation in earthworms and reduced reproductive performance, supporting a particle-mediated exposure pathway in soil	Pristine PVC, relatively high doses, one soil and organism, and no comparison with aged or environmentally coated particles
Saha et al. (2024), yard-waste compost	Plastic-associated legacy and precursor PFAS	Field sampling and vertical profiling of compost piles	Demonstrated PFAS occurrence on plastic materials and variations with composting stage and pile depth, providing field evidence of shared occurrence and redistribution	The observational design does not isolate adsorption, desorption, or causal PFAS–plastic transport mechanisms

PS: polystyrene; PTFE: polytetrafluoroethylene; PA: polyamide; PVC: polyvinyl chloride; MP: microplastic; NP: nanoplastic; PFOA: perfluorooctanoic acid; PFOS: perfluorooctane sulfonate; PFPeA: perfluoropentanoic acid; PFHxS: perfluorohexane sulfonate; FTSA: fluorotelomer sulfonic acid; GenX: hexafluoropropylene oxide dimer acid

The core studies do not support a universal conclusion that MPs and NPs increase PFAS mobility. Instead, they show three possible outcomes. Plastic particles may retard PFAS when strongly sorbed compounds are carried by particles that are retained in the porous medium. They may facilitate transport when the PFAS–particle complex remains stable and the particle is mobile. They may also have little measurable effect when PFAS remains predominantly dissolved or when soil and sediment surfaces exert stronger control than the plastic phase (Rong et al., 2023; Xie et al., 2025; Xu et al., 2024).

A similar conditional response is evident for adsorption. Polyamide particles displayed high intrinsic affinity for several PFAS, but much of this capacity was suppressed after contact with soil because mineral and biological coatings covered the polymer surface (Sun et al., 2024). This result is particularly important because it shows that adsorption measured on isolated plastic does not represent the effective adsorption of the same material after incorporation into a soil matrix.

The available biological evidence is even narrower. The earthworm experiment by Sobhani et al. (2021) demonstrated that PFAS-bearing MPs can increase internal exposure and reproductive effects, but no comparable studies have evaluated other soil invertebrates, microorganisms, crop plants, predators, or trophic transfer. Field evidence is similarly limited. The compost study provides evidence of PFAS associated with plastic materials under realistic conditions, but it cannot determine whether the observed distributions resulted from adsorption, incorporation during manufacturing, precursor transformation, or independent contaminant migration (Saha et al., 2024).

The main conclusion emerging from the core evidence is therefore not that plastic particles consistently act as PFAS vectors, but that their influence is conditional on particle mobility, surface condition, PFAS structure, and competition with the surrounding matrix. The following sections compare the core studies with complementary mechanistic evidence to determine when these interactions are likely to affect PFAS retention, transport, and biological exposure in soils.

### Adsorption and desorption in soil-relevant matrices

PFAS adsorption onto MPs and NPs is governed by the combined properties of the fluorinated compound, the polymer surface, and the surrounding environmental matrix. The available studies show that polymer identity, PFAS chain length, terminal functional group, particle size, surface oxidation, and solution chemistry influence the magnitude and reversibility of adsorption. Consequently, adsorption capacity measured for an isolated plastic should not be interpreted as an intrinsic and constant property of that polymer under environmental conditions (Freilinger et al., 2025; Hatinoglu et al., 2023; Llorca et al., 2018; Wang et al., 2015).

Polymer chemistry is a primary source of variation. Nonpolar polymers such as PE, PP, and PS interact with PFAS mainly through hydrophobic partitioning and dispersion forces, although surface charge and pore structure can also influence uptake. Polar polymers provide additional interaction domains. Freilinger et al. (2025), in a comparison involving 18 polymers and eight PFAS, observed markedly greater adsorption by PA materials than by PE. Computational results supported the experimental observations and indicated stronger interaction energies for PFAS associated with amide-containing polymer structures. Similarly, the combined experimental and theoretical analysis of PFOS adsorption onto PP-NPs identified dispersion forces as the main stabilizing contribution, with additional electrostatic and polar interactions (Simonetti et al., 2025).

PFAS structure modifies these polymer-dependent responses. Increasing fluorocarbon chain length generally strengthens association with hydrophobic surfaces, while the terminal functional group and ionization state control electrostatic and polar interactions. Wang et al. (2015) found greater partitioning of neutral perfluorooctanesulfonamide (FOSA) than anionic PFOS onto PE, PS, and PVC, despite the compounds having similar fluorocarbon chain lengths. Llorca et al. (2018) also observed that sulfonates and sulfonamides generally showed greater adsorption than several carboxylates and that PS-based particles exhibited higher affinity than HDPE under the tested conditions. These differences indicate that results obtained for PFOA or PFOS alone cannot represent the behavior of the wider PFAS class.

The only core study that directly compared isolated MPs, soil, and a soil–MP mixture provides important evidence of matrix interference. Sun et al. (2024) found that isolated PA-MPs showed substantially greater adsorption of PFOA, PFHxS, 6:2 FTSA, and GenX than the studied paddy soil. The adsorption capacity followed the general order PA-MPs ≫ PA-MP–soil mixture > soil. However, mixing the particles with soil reduced their PFAS adsorption capacity by approximately 7–108-fold compared with isolated PA-MPs. Depending on PFAS identity and MP dosage, the inhibition of adsorption ranged from approximately 22 to 97% (Sun et al., 2024).

This reduction was attributed to competition between PFAS and soil constituents for active sites on the PA surface, together with the physical coverage of the particles by soil material. Scanning electron microscopy showed that initially smooth PA particles became rough and coated with soil particles shortly after contact with the matrix. Biofilm-like material was also visible after longer conditioning periods. Although these coatings reduced the adsorption capacity of the isolated plastic, the addition of PA-MPs still increased the total PFAS retention of the soil–plastic mixture relative to soil alone. MPs may therefore contribute additional adsorption domains without retaining the high adsorption capacity measured in particle-only systems (Sun et al., 2024).

The kinetics reported by Sun et al. (2024) also illustrate why equilibrium values from isolated particles may not describe soil systems adequately. Most PFAS adsorption onto PA-MPs occurred during the first hours, and equilibrium was reached after approximately 24 h for isolated PA and soil. In the PA-MP–soil mixture, equilibrium required approximately 96 h. The longer equilibration period indicates that the soil matrix introduced mass-transfer restrictions and competing adsorption domains that were absent from the isolated polymer system. These processes are likely to become more important in structured soils, where diffusion through aggregates and access to internal adsorption sites occur over longer time scales.

Complementary studies using secondary or environmentally altered plastics confirm that the original polymer surface does not remain unchanged after release. Salawu et al. (2024) showed that PFAS adsorption onto secondary PET particles generated from bottles was spontaneous and reached equilibrium within several hours. Adsorption decreased with increasing pH and natural organic matter concentration, whereas greater ionic strength favored uptake by reducing electrostatic repulsion. These results indicate that PFAS association with environmental MPs can respond rapidly to changes in pore-water chemistry, although the direction of the response depends on both PFAS speciation and particle charge.

Weathering may increase adsorption by producing cracks, pores, greater roughness, and oxygen-containing functional groups. Naturally aged PS-MPs showed higher partition coefficients for several legacy and emerging PFAS than virgin particles. Some compounds that were not measurably adsorbed onto virgin PS were detected on aged particles, suggesting that aging introduced additional adsorption domains or modified electrostatic constraints (Barhoumi et al., 2024). Artificially aged particles have also shown altered PFAS affinity, although the magnitude and direction of the change differed among polymers and aging procedures (Ning et al., 2024).

Biofilms represent another environmental modification of the particle surface. Bhagwat et al. (2021) found that biofilm-covered PE, PP, polyester, and PA microfibers adsorbed more PFOS than their virgin counterparts. Biofilm development also reduced and delayed PFOS desorption, indicating that extracellular polymeric substances may provide additional retention domains. Similar effects were observed for PFOA on biofilm-associated MPs, where proteins, polysaccharides, and oxygen-containing functional groups contributed to adsorption (Song et al., 2025). Field-incubated plastics likewise accumulated more PFAS than clean particles exposed under simplified laboratory conditions, supporting the influence of associated organic and inorganic material (Scott et al., 2021).

The results obtained with biofilms do not contradict the reduction in PA adsorption observed after contact with soil. Instead, they show that environmental coatings can act in different ways. A coating dominated by organic matter or extracellular polymeric substances may add PFAS-binding sites. A coating dominated by minerals, strongly competitive organic compounds, or tightly attached soil particles may mask the polymer surface and restrict access to internal domains. Natural soils commonly contain both types of material, and the effective outcome depends on their relative abundance, composition, and arrangement on the particle surface (Scott et al., 2021; Sun et al., 2024).

Natural organic matter also produces apparently conflicting responses. Pre-exposure of MPs to organic matter has increased PFAS uptake in some experiments by creating additional partitioning domains, whereas dissolved organic matter has reduced adsorption in others through site competition, particle stabilization, and electrosteric repulsion (Ateia et al., 2020; Salawu et al., 2024). These differences show that “organic matter” should not be treated as a single experimental variable. Its molecular composition, concentration, dissolved or particle-associated state, and contact time with the plastic determine whether it enhances or suppresses PFAS retention.

Desorption remains much less studied than adsorption. Most investigations determine equilibrium uptake under constant laboratory conditions but do not evaluate whether PFAS remain associated with the plastic after changes in pH, ionic strength, organic matter, particle aging, or biological ingestion. The lower PFOS desorption from biofilm-covered fibers observed by Bhagwat et al. (2021) indicates that environmental conditioning can prolong retention. However, comparable data for soil-coated particles, aged MPs in terrestrial matrices, short-chain PFAS, and emerging alternatives remain scarce.

This lack of desorption data limits the interpretation of MPs and NPs as environmental vectors. High adsorption capacity does not necessarily imply enhanced contaminant transport. If the particle is retained in the soil, strong adsorption may reduce PFAS mobility. Conversely, moderate and reversible adsorption onto a mobile particle may be more relevant for redistribution and exposure than high but effectively irreversible retention. The environmental role of plastics should therefore be evaluated using coupled information on adsorption, desorption, particle mobility, and competition with the soil matrix rather than adsorption capacity alone.

Three interaction regimes emerge from the available evidence. In a plastic-dominated regime, the polymer provides a substantial fraction of the available adsorption sites, as may occur with abundant PA particles or NPs with high specific surface area. In a matrix-dominated regime, minerals and soil organic matter control PFAS partitioning and the plastic contributes little to the overall mass balance. In a coating-mediated regime, the original polymer surface is replaced functionally by biofilms, organic matter, or mineral deposits, which may either increase or suppress PFAS retention. Natural soils are likely to shift among these regimes over time as particles weather, become coated, aggregate, and move through different soil horizons.

The current evidence therefore supports PFAS adsorption onto a wide range of MPs and NPs but does not support the assumption that isolated-particle adsorption capacities can be transferred directly to soils. The principal uncertainty is not whether adsorption occurs, but whether plastic-associated adsorption is large enough, persistent enough, and sufficiently coupled to particle movement to alter PFAS fate relative to the dominant soil phases.

### Cotransport, retention, and the conditional vector effect

The description of MPs and NPs as PFAS vectors requires more than evidence of adsorption. Particle-mediated transport can occur only when PFAS remain associated with the plastic during movement and when the particle itself can migrate through the porous medium. Adsorption onto a retained particle may instead delay PFAS transport, while weak association with a highly mobile particle may have little effect because most PFAS remains in the dissolved phase. The vector effect is therefore conditional on the balance among PFAS–particle affinity, particle mobility, desorption kinetics, and retention by the soil matrix.

The available column studies also show that the interaction is reciprocal. PFAS can change the surface charge, aggregation, and deposition of plastic particles, while the particles may simultaneously change PFAS partitioning and breakthrough. These two directions of influence should be evaluated separately because greater MP/NP mobility does not necessarily imply greater PFAS mobility.

Rong et al. (2023) demonstrated this distinction using negatively and positively charged PS-MPs in saturated quartz-sand columns. PFOA adsorption made the surface potential of the negatively charged particles less negative, reducing electrostatic repulsion with the sand and increasing their retention. In contrast, PFOA reduced the positive charge of aminated particles, increasing electrostatic and steric repulsion from the negatively charged collector surface and promoting their transport. Despite these opposite responses, both particle types reduced PFOA transport because the MPs were less mobile than dissolved PFOA. Thus, adsorption onto a particle did not generate transport facilitation but transferred part of the PFOA mass to a more strongly retained phase (Rong et al., 2023).

Solution chemistry can reverse the direction of the interaction. Zhang et al. (2025) found that PS-MPs generally inhibited PFOA transport in neutral and alkaline quartz-sand systems. PFOA adsorbed onto the particles through predominantly hydrophobic interactions, and particle deposition reduced the mobility of the associated fraction. Increasing ionic strength compressed the electrical double layer and reduced repulsion between PS-MPs and quartz, while Ca^2+^ produced stronger retention than Na^+^ through charge neutralization and possible cation bridging. Under these conditions, the presence of MPs increased PFOA retention (Zhang et al., 2025).

A different response occurred at pH 5. Acidification reduced the negative charge of the quartz surface and increased the retention of both dissolved PFOA and PS-MPs. When the contaminants were introduced together, however, competition between PFOA and MPs for attachment sites reduced the retention of PFOA relative to the single-solute condition. The particles therefore facilitated PFOA breakthrough under this particular condition, even though acidic conditions were unfavorable for MP transport. This result shows that apparent cotransport may arise from competitive occupation of the porous-medium surface rather than from the physical carriage of PFAS on mobile particles (Zhang et al., 2025).

PFOA also altered PS-MP transport by increasing the negative charge of the particles and reducing their aggregation under neutral conditions. The resulting increase in repulsion between the particles and quartz favored MP breakthrough. At low pH, this effect became weak because proton-induced compression of the electrical double layer promoted aggregation and attachment. Accordingly, the influence of PFAS on particle mobility depends on whether surfactant adsorption or the background solution chemistry exerts the stronger control over the particle surface (Zhang et al., 2025).

PFAS molecular structure represents another control on particle transport. Zhao et al. (2025a) compared PTFE-MP transport in the presence of long-chain PFOA and short-chain PFPeA. PTFE recovery was higher with PFOA, which was attributed to its stronger association with the polymer and the greater steric hindrance created after adsorption. This surface modification reduced particle–particle aggregation and increased transport relative to PFPeA. Nevertheless, PTFE mobility decreased as pH declined and as ionic strength or cation valence increased, confirming that PFAS-induced stabilization remains constrained by electrolyte conditions (Zhao et al., 2025a).

The experiments with quartz sand isolate electrostatic and colloidal processes, but natural sediments introduce particle-size heterogeneity, mineral surfaces, and physical filtration. Xu et al. (2024) found that small PS particles, approximately 0.13 μm, showed high breakthrough in saturated sediment columns, whereas larger particles were retained more strongly. The principal explanation was physical straining, together with attachment to heterogeneous collector surfaces. PFOA increased the negative charge and transport of MPs at an influent pH of 4.7, particularly for the larger particles, but produced little change at pH 6.0. The sediment buffered the solution toward approximately neutral conditions, reducing the effect expected from the influent pH alone (Xu et al., 2024).

This buffering response illustrates an important limitation of laboratory interpretations based exclusively on the chemistry of the injected solution. In natural matrices, pH, ionic composition, and dissolved constituents may be modified during passage through the porous medium. The resulting transport behavior reflects the chemistry established within the column rather than the nominal composition of the influent.

Particle size also determined whether plastics changed PFOA transport in the experiments of Xie et al. (2025). The 0.1 μm PS-NPs were highly mobile and had no significant effect on PFOA recovery. In this case, both dissolved PFOA and the possible NP–PFOA complexes passed readily through the sediment, and the particle-associated fraction was insufficient to change total PFOA breakthrough. In contrast, 5 μm PS-MPs were retained more strongly and caused a small increase in PFOA retention. More than 91% of the PFOA remained unbound to the MPs, but the small associated fraction was transported with particles that were less mobile than the dissolved compound (Xie et al., 2025).

The comparison between NPs and MPs therefore does not support the simple assumption that greater surface area necessarily produces a stronger effect on PFAS transport. A small NP may have high adsorption potential but also high mobility, low residence time, or limited PFAS loading at environmentally relevant concentrations. A larger MP may adsorb less PFAS per unit mass but cause measurable retardation because it is efficiently retained by straining or attachment. Particle size affects both adsorption and transport, and these two effects may operate in opposite directions.

Three transport outcomes can be distinguished from the available evidence. Facilitated transport occurs when PFAS–particle association is maintained and the particle remains more mobile than the PFAS would be in the absence of plastics, or when competition with MPs reduces PFAS retention on the porous matrix. Retardation occurs when PFAS associate with particles that are preferentially deposited, aggregated, or physically strained. A negligible effect occurs when PFAS adsorption onto plastics is weak, when the associated fraction is small relative to the dissolved fraction, or when PFAS and particles have similarly high mobility.

These outcomes are not fixed properties of a polymer–PFAS pair. Changes in pH, ionic strength, cation valence, PFAS concentration, particle loading, surface charge, particle size, and collector properties can shift the same system from facilitation to retardation. This variability explains why the terms “carrier” and “vector” should not be used as synonyms for increased environmental mobility.

The environmental applicability of the current transport evidence remains restricted. The column studies were conducted under saturated flow, used short injection periods, and generally employed pristine spherical particles and PFAS concentrations substantially above those usually detected in uncontaminated soils. Even the natural-sediment studies used homogenized and sieved material, and quartz sand was added to maintain hydraulic conductivity in some experiments (Xie et al., 2025; Xu et al., 2024). Such procedures reduce the aggregation, macroporosity, layering, root channels, and preferential flow that characterize intact soil profiles.

Unsaturated conditions require particular attention. PFAS may accumulate at air–water interfaces, while MPs/NPs may be retained at solid–water–air contact lines or redistributed during wetting and drying. Preferential flow can bypass reactive soil domains, whereas periods of low flow can increase contact time, adsorption, aggregation, and biofilm development. None of the core cotransport studies reproduced these coupled processes over environmentally relevant time scales.

The present evidence therefore demonstrates that PFAS and MPs/NPs can alter each other’s transport, but it does not establish a consistent increase in contaminant migration. In most examined systems, the effect depended more strongly on particle retention and porous-medium properties than on adsorption capacity alone. Predicting the role of MPs/NPs in soils will require experiments that couple environmentally relevant adsorption with intact soil structure, variable saturation, preferential flow, aging, and long-term desorption.

### Bioaccumulation, toxicity, and ecological implications

The biological consequences of PFAS–MP/NP interactions depend on whether association with the plastic changes the contaminant fraction available for uptake. Adsorption may reduce exposure by decreasing the freely dissolved PFAS concentration, but it may also create an additional uptake route when contaminated particles are ingested, retained on biological surfaces, or internalized. The resulting effect depends on the relative contributions of dissolved uptake, particle uptake, and PFAS desorption after contact with the organism.

Direct terrestrial evidence is currently restricted to a single core study. Sobhani et al. (2021) exposed earthworms to soils containing PVC-MPs, PFOA, and PFOS and found that the presence of the particles increased PFAS bioaccumulation factors by up to 200%. Exposure to 500 and 1000 mg kg^−1^ PVC-MPs also reduced juvenile production, particularly when the particles occurred together with PFAS. The results indicate that ingested MPs can provide an additional route of PFAS exposure in soil organisms and that increased internal concentration may be accompanied by chronic reproductive effects.

The interpretation of this experiment requires caution. The PVC particles were pristine, the MP concentrations were high, and only one soil and one organism were tested. It is unclear whether the same response would occur with aged particles whose surfaces had been covered by minerals, organic matter, or biofilms. These coatings could reduce PFAS loading, alter particle ingestion, or modify desorption in the digestive tract. The study therefore demonstrates a possible mechanism rather than a general response for terrestrial invertebrates.

Evidence from plant systems shows that PFAS adsorption onto plastics can produce effects in opposite directions. Liu et al. (2023) observed that adsorption of PFOS onto PS-MPs and PS-NPs reduced the freely available PFOS fraction in a hydroponic soybean (*Glycine max*) system. This reduction attenuated oxidative stress and other acute phytotoxic responses. At the same time, PFOS altered the particle surface and increased the internalization of PS-NPs by plant tissues. Thus, lower PFAS bioavailability occurred together with greater NP uptake, demonstrating that the biological behavior of the two contaminants cannot be inferred from PFAS concentration alone.

Chen et al. (2024) reported a different pattern in *Eichhornia crassipes*. Co-exposure to PS-MPs increased the whole-plant bioconcentration factors of PFOA, PFOS, GenX, and F-53B, although the magnitude of the response differed among compounds. The combined toxicity was antagonistic for PFOA and PFOS but synergistic for GenX and F-53B. These contrasting responses indicate that greater PFAS accumulation does not necessarily result in the same type or intensity of physiological effect for legacy and alternative compounds. PFAS molecular structure, translocation within the plant, and the biological targets affected by each compound remain important controls. Additional experiments with floating macrophytes showed that hybrid MPs and PET-NPs can modify PFAS bioaccumulation and phytotoxicity, with the direction and intensity of the response depending on particle characteristics and the fluorinated compound considered (Chen et al., 2025; Li et al., 2025).

Both plant studies were conducted in water-based systems and cannot be transferred directly to crops grown in soil. In soil, root exposure is modified by adsorption to minerals and organic matter, rhizosphere chemistry, aggregate structure, microbial transformation, and the physical retention of MPs/NPs. The fraction of particle-associated PFAS that reaches the root surface may therefore be considerably smaller than in hydroponic exposure. Nevertheless, these studies demonstrate two mechanisms that should be examined in terrestrial plants: reduction of the dissolved PFAS fraction through adsorption and particle-mediated delivery to root tissues.

Supporting evidence from animals also shows that the direction of the combined response depends on particle properties and exposure conditions. Gupta et al. (2024) found that PFOS sorbed onto PS-MPs intensified developmental and neurobehavioral effects in zebrafish, including delayed hatching, increased malformation and mortality, oxidative stress, reduced acetylcholinesterase activity, and changes in neurotransmitter levels. Particle accumulation on the chorionic membrane was identified as an additional physical stressor that may have contributed to impaired gas exchange. The combined effect therefore involved both chemical transfer and direct particle-related disturbance.

Farajizadeh et al. (2025) demonstrated that PS-NPs increased PFOA uptake by Pacific oysters. At the highest tested NP concentration, 500 nm particles increased PFOA uptake approximately 2.3-fold, whereas 20 nm particles produced an increase of approximately 3.2-fold. The smaller particles also caused a stronger increase in PFOA-associated oxidative stress. These results support the relevance of particle size, because smaller NPs combine greater specific surface area with a higher probability of contact with and passage across biological interfaces.

Surface charge can be equally important. Zhang et al. (2024) evaluated negatively and positively charged PS-NPs associated with F-53B in aquatic insect larvae (*Chironomus kiinensis*). Both particle types increased adverse effects when the dissolved F-53B concentration was held constant, but positively charged NPs caused greater bioaccumulation, growth inhibition, oxidative stress, inflammation, and gut microbial disturbance. The stronger response was related to greater F-53B adsorption and to the affinity of positively charged particles for negatively charged biological membranes and microbial surfaces.

These results show that the concentration of PFAS associated with the particle is not the only relevant variable. The bioavailable fraction of particle-bound PFAS, particle adhesion to biological surfaces, cellular internalization, and retention in the digestive system may determine whether adsorption results in increased exposure. Two particles carrying the same PFAS mass may therefore produce different biological responses if their sizes, charges, or surface coatings differ.

Environmental organic matter further complicates these interactions. Junaid et al. (2024) observed that the combination of PS-NPs, F-53B, humic acid, and bovine serum albumin produced greater chronic toxicity in adult zebrafish than exposure to NPs, F-53B, or their binary mixture. The response included liver and intestinal damage, oxidative stress, changes in inflammatory and apoptotic pathways, and disruption of the gut microbiota. In this case, the organic corona did not provide a protective effect but altered the interfacial behavior of the particles and increased mixture toxicity.

The available evidence therefore does not support a uniform classification of PFAS–MP/NP toxicity as synergistic or antagonistic. Adsorption can reduce the dissolved concentration and attenuate short-term toxicity, as observed in soybean sprouts, or increase uptake and adverse effects, as observed in earthworms, oysters, aquatic insects, and some plant and fish experiments. In other cases, accumulation may increase while the toxic response remains antagonistic because the compounds act through different physiological pathways or because adsorption limits the fraction reaching sensitive tissues.

The distinction between toxicokinetic and toxicodynamic interactions is important. An apparent increase in toxicity may result from greater uptake and internal dose rather than from a direct interaction between PFAS and plastics at the target tissue. Conversely, antagonism may reflect reduced PFAS bioavailability without indicating that the plastic itself is harmless. Studies should therefore measure dissolved and particle-associated PFAS, particle uptake, tissue concentrations, desorption under biological conditions, and biological endpoints within the same experimental design.

Evidence for trophic transfer remains particularly weak. Uptake by earthworms and plants identifies possible entry points into terrestrial food webs, but no core study has quantified the subsequent transfer of plastic-associated PFAS to predators, herbivores, or higher trophic levels. The aquatic insect study of Zhang et al. (2024) is relevant because insects can connect aquatic and terrestrial food webs, but it did not directly quantify PFAS–NP transfer to consumers. Current evidence therefore indicates a plausible pathway rather than demonstrated trophic magnification caused by plastic particles.

The ecological interpretation is also constrained by the concentrations and exposure systems used in most studies. Experiments commonly apply pristine and uniform particles at concentrations above those expected in many environmental settings, together with a limited number of legacy or alternative PFAS. Short exposure periods may identify acute biochemical responses but do not represent the chronic and intermittent exposure expected in soils. Long-term studies are needed to determine whether particle-associated PFAS remain bioavailable after aging, coating, aggregation, and passage through soil profiles.

Taken together, the available studies show that MPs and NPs can modify PFAS uptake and toxicity, but the direction and magnitude of the response are conditional. Particle size, charge, polymer composition, surface conditioning, PFAS structure, exposure route, and organism physiology jointly determine the outcome. For terrestrial risk assessment, the main unresolved question is whether plastic-mediated exposure contributes substantially to internal PFAS doses relative to pore water, food, soil particles, and other environmental phases under realistic conditions.

### From controlled experiments to environmentally realistic soils

Controlled laboratory systems are necessary for identifying individual mechanisms, but their results describe potential interactions rather than their relative importance in natural soils. Experiments using clean particles, a single PFAS, deionized water, or quartz sand can isolate hydrophobic interactions, surface charge effects, cation bridging, aggregation, and attachment. In soils, these processes occur simultaneously with adsorption to organic matter and minerals, physical retention within pores, biological colonization, and changes in pore-water chemistry. The principal challenge is therefore to determine whether a mechanism that is detectable under controlled conditions remains strong enough to alter PFAS mass distribution, mobility, or biological exposure in the presence of competing soil processes.

The condition of the plastic particles is one of the clearest differences between laboratory and environmental systems. Commercial MPs and NPs usually have uniform size, smooth surfaces, and defined functional groups. Environmental particles are irregular, contain additives and fillers, and undergo oxidation, abrasion, fragmentation, and biological colonization. Weathering can create cracks and oxygen-containing groups that increase PFAS affinity, as observed for naturally aged PS particles (Barhoumi et al., 2024). However, contact with soil may also reduce access to the polymer surface through mineral deposition and biofilm formation, as demonstrated for PA-MPs incorporated into soil (Sun et al., 2024). Aging should therefore be treated as a change in the available interaction mechanisms rather than as a process that invariably increases adsorption.

The contrast between Scott et al. (2021) and Sun et al. (2024) illustrates this dependence on the surrounding matrix. Field-incubated plastics containing associated organic and inorganic material accumulated substantially more PFAS than clean plastics exposed in laboratory water (Scott et al., 2021). By contrast, PA particles that showed high intrinsic PFAS adsorption lost much of this capacity after contact with soil because their surfaces became covered by soil constituents (Sun et al., 2024). These findings are not contradictory. Organic-rich coatings may provide additional partitioning domains, whereas mineral-dominated coatings may mask the original polymer surface or intensify competition with the soil matrix.

Experimental concentration is another source of uncertainty. Elevated PFAS and plastic concentrations improve analytical detection and facilitate the construction of adsorption isotherms or breakthrough curves. They may also increase particle–PFAS contact, saturate competing soil sites, promote aggregation, or create a particle-associated fraction that would be negligible at field concentrations. A statistically significant interaction at high concentrations does not establish that the same process contributes meaningfully to PFAS transport or exposure in less contaminated soils.

The use of nominal concentrations also limits interpretation. PFAS may be lost through adsorption to experimental containers, retained on filters, associated with dissolved organic matter, adsorbed to the porous matrix, or bound to the plastic particles. MPs and NPs may likewise aggregate, attach to column materials, or settle during exposure. Studies that quantify only the initial and final dissolved concentrations cannot distinguish adsorption onto plastics from other loss processes. Reliable mass balances require separate measurements of dissolved PFAS, particle-associated PFAS, soil-associated PFAS, particle recovery, and experimental losses.

The experimental matrix strongly affects the apparent vector role of MPs and NPs. Quartz sand has low organic carbon content and relatively uniform surface properties, making plastic particles a more visible adsorption or transport phase. Natural soils contain organic matter, clays, metal oxides, carbonates, aggregates, and heterogeneous surface charges that may retain PFAS more strongly than the plastics present. Under these conditions, the plastic-associated fraction may represent only a small proportion of the total PFAS mass, even when the polymer has measurable affinity in isolated batch tests.

Soil structure introduces additional controls that are not reproduced in repacked columns. Intact soils contain macropores, root channels, aggregate boundaries, and layered horizons. Preferential flow may transport dissolved PFAS and small particles rapidly through selected pathways, while diffusion into aggregates and attachment within smaller pores can prolong retention. Repacking and homogenization remove much of this structure, reduce spatial variability, and alter the contact between contaminants and reactive surfaces. Results from such systems are mechanistically informative but cannot represent the full range of travel times and retention domains in field soils.

The dominance of saturated-flow experiments is also problematic. In the vadose zone, PFAS can accumulate at air–water interfaces, and plastic particles may be retained at solid–water–air contact lines. Wetting and drying change ionic concentration, interface area, particle aggregation, and the accessibility of adsorption sites. These transient conditions may control long-term redistribution more strongly than the steady saturated flow used in most column studies (Kookana et al., 2023).

Time scale further separates laboratory and environmental behavior. Most experiments last from hours to several days, whereas plastic particles and PFAS may remain in soils for years or decades. During this period, polymer oxidation, precursor transformation, biofilm maturation, mineral deposition, aggregation, and repeated adsorption–desorption cycles can change both particle mobility and PFAS affinity. Short experiments characterize initial interactions but provide little information on whether they persist, weaken, or reverse after prolonged environmental conditioning.

Field studies are essential for assessing these processes but remain scarce. The occurrence of PFAS and MPs in landfill–wastewater systems, biosolids, and compost confirms that the contaminants share sources and can reach terrestrial environments together (Ma et al., 2025; Prada et al., 2024; Saha et al., 2024). However, co-occurrence does not demonstrate that PFAS are transported by plastic particles. Field investigations must distinguish PFAS adsorbed after environmental release from fluorinated compounds originally incorporated into the plastic or produced through precursor transformation.

This distinction is particularly relevant for textiles and fluorinated polymers. Fibers released from treated fabrics may contain residual or transformation-derived PFAS before entering the environment (Schellenberger et al., 2019; Zhao et al., 2025b, 2025c). Detection of PFAS on such particles cannot be attributed automatically to environmental adsorption. Source characterization, polymer analysis, precursor measurements, and controlled desorption experiments are required to determine whether the particle is acting as a source, a sorbent, or both.

The same caution applies to biological effects. Increased toxicity during combined exposure may result from higher PFAS uptake, direct particle toxicity, physical obstruction, altered microbial communities, or independent effects of both contaminants. Demonstrating a plastic-mediated increase in PFAS risk requires evidence that the particle changes the internal PFAS dose or its distribution among tissues. Combined exposure alone is not sufficient to establish a carrier effect.

Four comparisons should guide the interpretation of future studies: pristine versus environmentally aged particles, homogeneous laboratory media versus intact natural soils, short-term breakthrough versus long-term retention, and apparent adsorption versus environmentally meaningful cotransport. These comparisons separate the existence of an interaction from its importance under field conditions.

As represented in Fig. 1, natural soils do not necessarily eliminate PFAS–MP/NP interactions. They can strengthen, suppress, or redirect them. Organic coatings and biofilms may increase PFAS retention, whereas mineral coverage and competitive adsorption may reduce access to the polymer. Preferential flow may promote particle movement through macropores, while aggregation and straining may immobilize the same particles in smaller pores. The environmental outcome depends on which of these processes dominates at a given location and time.

The available literature therefore supports a conditional rather than universal vector effect. MPs and NPs are most likely to alter PFAS fate when they are sufficiently abundant, have accessible adsorption domains, retain PFAS during movement, and remain mobile relative to the surrounding soil phases. When these conditions are not met, plastics may function primarily as localized reservoirs or may have little influence on the overall PFAS mass balance. This distinction should form the basis for interpreting laboratory results, designing field studies, and incorporating plastic particles into PFAS risk and transport models.

## Research gaps and priorities for future studies

### Environmental occurrence and field association

The first priority is to establish whether PFAS–MP/NP associations occur at environmentally relevant levels in soils. Current field evidence mainly demonstrates co-occurrence in compost, biosolids, landfill leachate, and wastewater-related residuals, but rarely measures the fraction of PFAS that is directly associated with plastic particles (Ma et al., 2025; Prada et al., 2024; Saha et al., 2024). Future field studies should quantify PFAS in pore water, bulk soil, soil organic matter, mineral fractions, and isolated plastic particles within the same sampling design.

The origin of PFAS detected on environmental plastics must also be determined. PFAS associated with a particle may have been adsorbed after release, incorporated during manufacturing, generated through precursor transformation, or deposited together with organic and mineral coatings. This distinction is particularly important for textile fibers, fluorinated polymers, and multilayer materials that may already contain PFAS before entering the environment (Schellenberger et al., 2019; Zhao et al., 2025c).

Field sampling should include vertical soil profiles, different land uses, seasonal conditions, and known contamination sources. Agricultural soils receiving biosolids or compost, areas affected by aqueous film-forming foams, landfill surroundings, industrial sites, and soils irrigated with reclaimed water are particularly relevant. Without spatially resolved measurements, the contribution of plastic particles to PFAS redistribution cannot be separated from independent movement of dissolved or soil-bound PFAS.

### Standardization and analytical quality control

Comparison among studies is currently limited by differences in particle size, polymer type, PFAS concentration, exposure time, water chemistry, and reporting units. A minimum experimental dataset should include particle number and mass, size distribution, morphology, polymer identity, surface charge, specific surface area, aging condition, additive content, and the presence of environmental coatings. PFAS should be reported individually rather than only as total concentrations, with clear information on chain length, functional group, purity, and initial speciation.

Experimental mass balances require substantial improvement. Studies should quantify PFAS in the dissolved phase, on the plastic particles, on soil or sediment surfaces, and in experimental losses such as containers and filters. Particle recovery should also be measured throughout batch, column, and biological tests. Without this information, an apparent reduction in aqueous PFAS cannot be attributed reliably to adsorption onto MPs/NPs.

These rigorous analytical requirements are further complicated by the ongoing technical bottlenecks in isolating and quantifying both the polymers and the contaminants within complex experimental systems. On one hand, isolating MPs/NPs from organic-rich matrices (such as soils, sediments, or biofilms) remains highly challenging, where extraction protocols often suffer from low recovery rates or risk altering the polymer structure, while the detection of sub-micron particles pushes the limits of current spectroscopic techniques. On the other hand, the analytical determination of PFAS is equally plagued by critical hurdles, most notably widespread background contamination from laboratory consumables and PTFE-containing equipment, which demands strict quality controls. Additionally, severe matrix effects during LC–MS/MS analysis caused by co-extracted organic matter often interfere with quantification, requiring advanced cleanup steps and mass-labeled internal standards.

Moreover, controls are needed to distinguish polymer effects from other adsorption phases. At minimum, experiments should include PFAS without plastic, plastic without PFAS, soil without plastic, and combined soil–plastic systems. When organic matter or biofilms are considered, dissolved and particle-associated controls should be evaluated separately. These comparisons are necessary because organic coatings may either increase the number of binding domains or mask the original polymer surface (Salawu et al., 2024; Scott et al., 2021; Sun et al., 2024).

Studies should also report environmentally relevant concentrations alongside higher concentrations used for mechanistic analysis. High-dose experiments may remain necessary for analytical detection and model fitting, but they should not be the sole basis for conclusions about field behavior. Testing across a concentration gradient would help determine whether the observed interaction persists when plastic and PFAS concentrations approach those found in terrestrial environments.

### Environmentally conditioned particles and intact soils

The use of pristine and monodisperse particles remains one of the principal limitations of the evidence base. Future experiments should include naturally weathered MPs/NPs or particles subjected to validated aging procedures that reproduce oxidation, surface cracking, additive loss, mineral adhesion, and biological colonization. Aging methods should be described in sufficient detail to permit comparison among studies.

Artificial aging alone is not sufficient. Particles should be conditioned directly in soils, compost, biosolids, or other terrestrial matrices before PFAS exposure. The comparison among pristine, artificially aged, and soil-conditioned particles would allow researchers to determine whether oxidation, biofilm formation, and mineral coatings exert independent or competing effects on PFAS adsorption and desorption (Barhoumi et al., 2024; Sun et al., 2024).

Experiments should move beyond quartz sand and homogenized sediment. Intact soil cores are needed to preserve aggregation, macropores, root channels, mineralogical variation, and preferential flow. Multiple soil types should be included, covering differences in texture, organic carbon, pH, clay mineralogy, iron and aluminum oxides, carbonate content, and cation-exchange capacity. Results from one low-organic-carbon soil cannot represent the range of terrestrial matrices.

Unsaturated flow and transient moisture conditions must receive greater attention. Most transport studies have been conducted under continuous saturation, although PFAS and plastic particles in the vadose zone encounter variable water contents, air–water interfaces, wetting and drying cycles, and changes in ionic concentration. These processes may modify aggregation, attachment, interfacial adsorption, and remobilization (Kookana et al., 2023).

### Long-term adsorption, desorption, and transport

The short duration of most experiments prevents evaluation of environmental persistence. Studies lasting several hours or days describe initial adsorption and transport but do not capture polymer oxidation, biofilm maturation, precursor transformation, mineral deposition, or repeated adsorption–desorption cycles. Longer experiments are needed to determine whether plastic-associated PFAS remain retained, become progressively immobilized, or return to pore water over time.

Desorption should be investigated with the same attention currently given to adsorption. Relevant scenarios include dilution, changes in pH or ionic strength, transfer between soil horizons, ingestion by organisms, passage through digestive fluids, and release during polymer degradation. A particle can only act as an effective exposure vector if PFAS remain associated during transport and become available at a new location or within an organism.

Column studies should compare saturated and unsaturated flow, constant and pulsed inputs, and homogeneous and preferential flow. Breakthrough curves should be accompanied by measurements of retained particle and PFAS distributions at different depths. These measurements would help distinguish true particle-mediated cotransport from competitive adsorption, remobilization of dissolved PFAS, and independent movement of the two contaminants.

The transport of NPs deserves specific attention, but size alone should not be treated as evidence of high mobility. NP transport depends on aggregation, surface charge, organic coatings, and attachment to soil colloids. Experiments should therefore measure particle-size distributions during transport rather than relying only on the nominal size supplied by the manufacturer (Xie et al., 2025; Xu et al., 2024).

### Chemical diversity and mixture effects

The current literature is dominated by PS particles and PFOA or PFOS. This narrow chemical range is insufficient for evaluating the diversity of plastics and PFAS present in soils. Future studies should compare common environmental polymers such as PE, PP, PVC, PET, PA, polyester fibers, and fluoropolymers, including irregular fragments and fibers rather than only spherical beads.

PFAS selection should include short- and long-chain PFCAs and PFSAs, fluorotelomer compounds, ether-based alternatives, neutral precursors, and mixtures occurring in contaminated sites. Comparisons should be based on chain length, functional group, charge, and precursor status. Emerging alternatives cannot be assumed to behave like PFOA or PFOS because their polymer affinity, mobility, transformation, and toxicological profiles may differ substantially (Freilinger et al., 2025; Llorca et al., 2018).

Mixture experiments should also consider non-PFAS co-contaminants. Metals, hydrocarbons, surfactants, nutrients, dissolved organic matter, and other emerging contaminants may compete for plastic and soil surfaces or alter particle aggregation. Binary PFAS–plastic systems provide limited information on environments where multiple substances coexist.

### Biological uptake and combined ecotoxicity

Combined ecotoxicity in soils remains one of the least developed areas of the field. Direct terrestrial evidence is currently limited to earthworms, and comparable information is lacking for microorganisms, nematodes, collembolans, other soil invertebrates, crop plants, and terrestrial predators (Sobhani et al., 2021). This gap prevents evaluation of whether plastic-mediated PFAS exposure affects soil functions, food production, or terrestrial food webs.

Future biological studies should measure both exposure and internal dose. Experimental designs should quantify dissolved PFAS, particle-associated PFAS, ingested or internalized plastics, tissue PFAS concentrations, and relevant physiological endpoints. Without tissue measurements, it is not possible to determine whether greater toxicity results from increased PFAS uptake, direct plastic effects, or independent action of both contaminants.

Plant studies should move from hydroponic systems to soils with contrasting properties. Root uptake, translocation to shoots and edible tissues, rhizosphere interactions, and the role of particle size should be assessed under realistic soil concentrations. These experiments are necessary to evaluate whether MPs/NPs modify dietary exposure to PFAS through crops.

Trophic transfer also requires direct testing. Uptake by earthworms, plants, or aquatic insects identifies a potential entry point but does not demonstrate transfer to higher consumers. Predator–prey studies should determine whether PFAS associated with MPs/NPs are retained, desorbed, or biomagnified during successive trophic steps.

Combined effects should not be classified as synergistic or antagonistic solely from external exposure concentrations. Studies should distinguish toxicokinetic effects, such as changes in uptake and elimination, from toxicodynamic effects at biological targets. This distinction is necessary because plastic adsorption may reduce dissolved PFAS exposure while simultaneously increasing particle ingestion or tissue delivery.

### Modeling, monitoring, and risk assessment

Current transport models generally describe advection, dispersion, attachment, and detachment under simplified conditions. Future models should incorporate PFAS adsorption and desorption kinetics, particle aggregation, surface aging, mineral and organic coatings, air–water interfacial retention, and competition with soil adsorption phases. Parameterization should be based on soil-specific experiments rather than values obtained only from water or quartz sand.

Models should also represent PFAS and plastic particles as interacting but distinct phases. Assuming permanent attachment between PFAS and MPs/NPs can overestimate cotransport, while ignoring reversible association can underestimate transient retention or remobilization. Coupled mass-balance approaches are needed to track dissolved, soil-bound, plastic-associated, and biologically accumulated PFAS.

Monitoring programs should combine PFAS analysis with standardized MP/NP characterization. Measuring one contaminant group without the other cannot determine whether shared sources or particle-associated transport are relevant. Long-term monitoring at contaminated sites would provide the evidence required to validate laboratory-derived mechanisms and transport models.

Until such data are available, the vector role of MPs/NPs should be treated as a site-specific hypothesis rather than a general property of plastic contamination. Risk assessments should consider plastic-mediated transport when particles are abundant, mobile, environmentally conditioned, and capable of retaining PFAS under local soil conditions. In other settings, organic matter, minerals, and dissolved transport may remain the dominant controls.

## Conclusion

Understanding PFAS–MP/NP interactions in soils is important because terrestrial matrices can simultaneously retain, transform, and redistribute both contaminant groups before their transfer to groundwater, surface water, plants, and soil organisms. The evidence reviewed here confirms that PFAS can associate with MPs and NPs through hydrophobic, electrostatic, polar, and dispersion interactions. However, adsorption onto a plastic particle does not necessarily result in enhanced PFAS mobility. The particle may facilitate transport, retard PFAS movement, or produce no measurable effect depending on PFAS affinity, particle mobility, desorption, and retention by the surrounding matrix.

The main conclusion of this review is that the PFAS vector effect of MPs and NPs is conditional rather than universal. Results obtained with pristine particles, high contaminant concentrations, homogeneous quartz sand, and controlled solution chemistry are useful for identifying mechanisms but cannot be transferred directly to natural soils. Weathering and biofilms may create additional adsorption domains, whereas mineral coatings, organic matter, competitive adsorption, aggregation, and physical straining may suppress or reverse the interactions observed in simplified systems. In many soils, the mineral and organic phases are likely to remain the dominant controls on PFAS partitioning, while plastics act as secondary reservoirs whose importance varies with local conditions.

The evidence on biological consequences is still insufficient for terrestrial risk assessment. The available soil study indicates that MPs can increase PFAS bioaccumulation and reproductive toxicity in earthworms, but comparable data are lacking for crop plants, soil microorganisms, other invertebrates, predators, and terrestrial food webs. Progress therefore depends on field measurements, intact-soil experiments, environmentally conditioned particles, realistic concentrations, complete PFAS and particle mass balances, long-term desorption and transport assessments, and direct measurements of internal exposure. Until such evidence becomes available, PFAS–MP/NP interactions should be evaluated as site-specific processes rather than assumed to increase contaminant mobility or ecological risk in every soil system.

## Supplementary Information

Below is the link to the electronic supplementary material.

Supplementary file1 (DOCX 116 KB)

## Data Availability

All data supporting the findings of this study are available within the paper.
