# Supplementary material for: Microplastic and nanoplastic interactions with per- and polyfluoroalkyl substances (PFAS) in soils: a critical review of main findings and knowledge gaps
Source: Environ Geochem Health. 2026 Aug 29;48(14):561. doi: 10.1007/s10653-026-03419-x (PMC13526050; doi:10.1007/s10653-026-03419-x)
Supplement: Supplementary file 1 [file 10653_2026_3419_MOESM1_ESM.docx]

**Supplementary Material**

**Microplastic and Nanoplastic Interactions with Per- and Polyfluoroalkyl Substances (PFAS) in Soils: A Critical Review of Main Findings and Knowledge Gaps**

Pedro Augusto Soares ^1^, Guilherme Silva de Souza ^1^, Valéria Guimarães Silvestre Rodrigues ^1^, Jacqueline Zanin Lima ^1^ 🖂

^1^ Department of Geotechnical Engineering, São Carlos School of Engineering, University of São Paulo - 400 Trabalhador São Carlense Ave. São Carlos, Brazil 13566-590

Jacqueline Zanin Lima (🖂)

e-mail: jacqueline.zanin.lima@usp.br

**Table SM1.** Conceptual definitions of environmental domains.

| Category | | Key aspects covered |
| --- | --- | --- |
| Health |  | This category includes studies that investigate the effects of PFAS and MPs/NPs on human organisms or on biological models relevant to human health. |
| Soil |  | This domain comprises studies analyzing the occurrence, transport, retention, sorption, or effects of PFAS and MPs/NPs in soil environments. |
| Water |  | This category includes publications addressing the presence, behavior, and fate of MPs/NPs and PFAS in aquatic systems, including both freshwater and marine environments. |
| Ecosystem |  | This domain includes studies evaluating the combined effects of PFAS and MPs/NPs considering environmental functioning as an integrated system. |
| Air |  | This category encompasses studies dealing with the presence and movement of PFAS and MPs/NPs in the atmosphere. |

**Table SM2.** Reference, original domain, PFAS, and main mechanism or process of the complementary studies addressing PFAS–MP/NP interactions.

| **Reference, title, and DOI** | **Original domain** | **PFAS / contaminant** | **Main mechanism or process** | **Complementary contribution to the review** |
| --- | --- | --- | --- | --- |
| Chen et al. (2025). The emerging threat of hybrid microplastics: Impacts on per(poly)fluoroalkyl substance bioaccumulation and phytotoxicity in floating macrophytes. Journal of Hazardous Materials. DOI: 10.1016/j.jhazmat.2025.139130 | Ecosystem | PFOA and GenX | Mixed-polymer effects on uptake and phytotoxicity | Showed that hybrid PP/PE/PS microplastics altered PFOA and GenX bioaccumulation and phytotoxicity in floating macrophytes. |
| Farajizadeh et al. (2025). Enhanced uptake of perfluorooctanoic acid by polystyrene nanoparticles in Pacific oyster (*Magallana gigas*). Comparative Biochemistry and Physiology C-Toxicology & Pharmacology. DOI: 10.1016/j.cbpc.2024.110119 | Ecosystem | PFOA | Particle-mediated bioavailability | Showed that polystyrene nanoparticles enhanced PFOA uptake in Pacific oysters, providing complementary evidence that particle association can alter internal exposure. |
| Freilinger et al. (2025). Interactions between perfluorinated alkyl substances (PFAS) and microplastics (MPs): Findings from an extensive investigation. Journal of Hazardous Materials Advances. DOI: 10.1016/j.hazadv.2025.100740 | Water | PFBA, PFOA, PFOS | Polymer chemistry, pH, and sorption kinetics | Compared a broad set of polymers and PFAS and showed particularly strong interactions with polyamide, supporting the role of polymer functional groups in PFAS retention. |
| Li et al. (2025). Exploring the phytotoxicity mechanisms of PET nanoplastics and 6:2 FTSA in water hyacinth under individual and combined exposure scenarios. Journal of Hazardous Materials. DOI: 10.1016/j.jhazmat.2025.137675 | Ecosystem | 6:2 FTSA | Combined phytotoxicity | Reported enhanced phytotoxicity, oxidative stress, and photosynthetic disruption under combined exposure to PET nanoplastics and 6:2 FTSA in water hyacinth. |
| Simonetti et al. (2025). Unveiling the adsorption mechanism of perfluorooctane sulfonate onto polypropylene nanoplastics: A combined theoretical and experimental investigation. Water Research. DOI: 10.1016/j.watres.2025.123324 | Water | PFOS | Molecular adsorption mechanisms | Combined experimental and theoretical approaches to explain PFOS association with polypropylene nanoplastics and the contribution of non-covalent interactions. |
| Song et al. (2025). Adsorption performance and mechanism of perfluorooctanoic acid on biofilms-attached microplastics. Journal of Water Process Engineering. DOI: 10.1016/j.jwpe.2025.108885 | Water | PFOA | Biofilm-mediated sorption | Examined PFOA adsorption onto biofilm-attached PE and PS, demonstrating that biological coatings create or modify sorption domains. |
| Tang et al. (2025). Molecular-Level Insights into the Influence of Natural Organic Matter on Nanoplastic-Small Molecule Emerging Contaminant Interactions. Langmuir. DOI: 10.1021/acs.langmuir.5c05153 | Water | PFOS | Natural organic matter and competitive interfacial interactions | Provided molecular-level evidence that natural organic matter can compete with or redirect PFOS interactions at nanoplastic surfaces. |
| Tumrani et al. (2025). Biodegradation of PLA in surface water and its behavior towards PFOA: interactional characteristics and computational insights. Colloid and Polymer Science. DOI: 10.1007/s00396-025-05423-6 | Water | PFOA | Biodegradation and surface transformation | Showed that PLA degradation generated oxygen-containing groups and substantially increased PFOA adsorption, illustrating changes during environmental exposure. |
| Zhao et al. (2025). Molecular-level insights into the secondary per-and poly-fluoroalkyl substance (PFAS) in durable water repellent fabric: Generation, properties, and adsorption mechanism. Colloids and Surfaces A-Physicochemical and Engineering Aspects. DOI: 10.1016/j.colsurfa.2025.137524 | Water | PFAS derived from DWR fluoropolymers | Secondary PFAS generation and re-adsorption | Clarified how durable water-repellent fabrics generate secondary PFAS and how released compounds can interact again with synthetic fibers. |
| Zhao et al. (2025). Research on the PFAS release and migration behavior of multi-layer outdoor jacket fabrics. Journal of Hazardous Materials. DOI: 10.1016/j.jhazmat.2025.137218 | Water | Short-chain fluorinated polymers, PFAAs, and n:2 FTOHs | Co-release from multilayer textiles | Documented the release and migration of PFAS and microplastic fibers during aging, washing, and abrasion of outdoor jacket fabrics. |
| Barhoumi et al. (2024). Insight into the effect of natural aging of polystyrene microplastics on the sorption of legacy and emerging per- and polyfluorinated alkyl substances in seawater. Heliyon. DOI: 10.1016/j.heliyon.2024.e40490 | Ecosystem | Legacy and emerging PFAS, including PFOS and PFOA | Natural aging and biofouling | Compared virgin and naturally aged polystyrene and showed that oxidation, surface alteration, and biofilm development modify sorption of legacy and emerging PFAS. |
| Bere et al. (2024). Role of Fluorocarbon Chain Length in the Adsorption of Perfluoroalkyl Substances on Nanoplastic Particles. ACS ES&T Water. DOI: 10.1021/acsestwater.4c00689 | Water | C4, C6, and C8 PFAS | PFAS chain length, charge, and aggregation | Showed that fluorocarbon chain length affects adsorption, surface-charge neutralization, and nanoplastic aggregation, which may modify particle mobility. |
| Chen et al. (2024). The bioaccumulation and ecotoxicity of co-exposure of per(poly)fluoroalkyl substances and polystyrene microplastics to *Eichhornia crassipes*. Water Research. DOI: 10.1016/j.watres.2024.121878 | Ecosystem | PFOA, PFOS, GenX, F-53B | Compound-specific plant uptake and ecotoxicity | Evaluated co-exposure to PS microplastics and four PFAS in Eichhornia crassipes and found compound-dependent changes in bioaccumulation and toxicity. |
| Gupta et al. (2024). Sorption of PFOS onto polystyrene microplastics potentiates synergistic toxic effects during zebrafish embryogenesis and neurodevelopment. Chemosphere. DOI: 10.1016/j.chemosphere.2024.143462 | Ecosystem | PFOS | Sorption-linked combined toxicity | Demonstrated that PFOS sorption onto polystyrene microplastics potentiated embryonic and neurodevelopmental toxicity in zebrafish. |
| Junaid et al. (2024). Exacerbated interfacial impacts of nanoplastics and 6:2 chlorinated polyfluorinated ether sulfonate by natural organic matter in adult zebrafish: Evidence through histopathology, gut microbiota, and transcriptomic analysis. Journal of Hazardous Materials. DOI: 10.1016/j.jhazmat.2024.135038 | Ecosystem | F-53B | Natural-organic-matter-mediated combined effects | Showed that natural organic matter intensified nanoplastic–F-53B interactions, bioaccumulation, and biological effects in adult zebrafish. |
| Liu et al. (2024). Co-accumulation characteristics and interaction mechanism of microplastics and PFASs in a large shallow lake. Journal of Hazardous Materials. DOI: 10.1016/j.jhazmat.2024.135780 | Water | PFBA, HFPO-DA, PFHxA | Field co-occurrence and particle-associated PFAS | Linked microplastic morphology and biofilm development with the distribution and sediment accumulation of selected PFAS in a large shallow lake. |
| Ning et al. (2024). Adsorption behaviors of perfluorooctanoic acid on aged microplastics. Water Environment Research. DOI: 10.1002/wer.11080 | Water | PFOA | Artificial aging and oxidation | Compared PFOA adsorption on aged PS, PE, and PET and showed that aging effects are polymer-specific and linked to changes in surface chemistry and morphology. |
| Prada et al. (2024). Microplastics and per- and polyfluoroalkyl substances (PFAS) in landfill-wastewater treatment systems: A field study. Science of The Total Environment. DOI: 10.1016/j.scitotenv.2024.176751 | Soil | Multiple PFAS and MPs | Field co-occurrence in waste-management systems | Measured MPs and PFAS across linked landfill and wastewater-treatment systems. It supports shared pathways and transfer to solid residuals but does not directly demonstrate a carrier mechanism. |
| Salawu et al. (2024). Adsorption of PFAS onto secondary microplastics: A mechanistic study. Journal of Hazardous Materials. DOI: 10.1016/j.jhazmat.2024.134185 | Water | Multiple PFAS | Weathering-sensitive sorption | Demonstrated PFAS adsorption onto secondary PET microplastics and showed that pH, ionic strength, and natural organic matter modify particle–PFAS affinity. |
| Zhang et al. (2024). Nanoplastics Affect the Bioaccumulation and Gut Toxicity of Emerging Perfluoroalkyl Acid Alternatives to Aquatic Insects (*Chironomus kiinensis*): Importance of Plastic Surface Charge. ACS Nano. DOI: 10.1021/acsnano.3c12009 | Ecosystem | F-53B | Surface-charge-dependent bioaccumulation and toxicity | Demonstrated that nanoplastic surface charge controlled the bioaccumulation and gut toxicity of F-53B in aquatic insects. |
| Brahana et al. (2023). Uptake and release of perfluoroalkyl carboxylic acids (PFCAs) from macro and microplastics. Environmental Science-Processes & Impacts. DOI: 10.1039/d3em00209h | Water | PFCAs | Reversible uptake and release | Evaluated PFCA uptake and release from polyethylene macro- and microplastics, supporting the interpretation of plastics as reversible reservoirs rather than permanent sinks. |
| Hatinoglu et al. (2023). Modified linear solvation energy relationships for adsorption of perfluorocarboxylic acids by polystyrene microplastics. Science of The Total Environment. DOI: 10.1016/j.scitotenv.2022.160524 | Water | PFCAs | Predictive sorption modelling | Developed a modified linear solvation energy relationship for PFCA adsorption onto polystyrene, providing a mechanistic framework for compound-specific sorption. |
| Liu et al. (2023). Polystyrene micro and nanoplastics attenuated the bioavailability and toxic effects of Perfluorooctane sulfonate (PFOS) on soybean (*Glycine max*) sprouts. Journal of Hazardous Materials. DOI: 10.1016/j.jhazmat.2023.130911 | Water | PFOS | Reduced bioavailability and toxicity | Showed that polystyrene micro- and nanoplastics attenuated PFOS bioavailability and toxicity in soybean sprouts, illustrating an antagonistic outcome. |
| Bhagwat et al. (2021). Biofilms Enhance the Adsorption of Toxic Contaminants on Plastic Microfibers under Environmentally Relevant Conditions. Environmental Science & Technology. DOI: 10.1021/acs.est.1c02012 | Ecosystem | PFOS and Pb | Biofilm effects on plastic microfibers | Reported increased PFOS adsorption on biofilm-covered microfibers, linking biofilm development to changes in surface area, hydrophobicity, and contaminant retention. |
| Scott et al. (2021). Perfluoroalkylated Substances (PFAS) Associated with Microplastics in a Lake Environment. Toxics. DOI: 10.3390/toxics9050106 | Water | Multiple PFAS | Field conditioning and acquired surface coatings | Found greater PFAS accumulation on field-incubated plastics than on clean laboratory particles, indicating that environmental coatings can redefine the effective sorption surface. |
| Ateia et al. (2020). Sorption behavior of real microplastics (MPs): Insights for organic micropollutants adsorption on a large set of well-characterized MPs. Science of The Total Environment. DOI: 10.1016/j.scitotenv.2020.137634 | Water | Multiple PFAS and organic micropollutants | Real-plastic composition and surface heterogeneity | Showed that additives, fillers, roughness, and organic coatings can alter PFAS sorption relative to chemically pure polymers. |
| Schellenberger et al. (2019). Release of Side-Chain Fluorinated Polymer-Containing Microplastic Fibers from Functional Textiles during Washing and First Estimates of Perfluoroalkyl Acid Emissions. Environmental Science and Technology. DOI: 10.1021/acs.est.9b04165 | Water | 6:2 FTOH and related PFAAs | Shared textile source and precursor emissions | Demonstrated the release of side-chain fluorinated polymer-containing microfibers during washing and estimated associated perfluoroalkyl acid emissions. |
| Llorca et al. (2018). Adsorption of perfluoroalkyl substances on microplastics under environmental conditions. Environmental Pollution. DOI: 10.1016/j.envpol.2017.12.075 | Water | 18 PFAS (PFCAs, PFSAs, and one sulfonamide) | Sorption/desorption and polymer-dependent partitioning | Quantified sorption of 18 PFAS by several microplastic types, showing that affinity depends on PFAS structure, polymer composition, and environmental conditions. |
| Wang et al. (2015). The partition behavior of perfluorooctanesulfonate (PFOS) and perfluorooctanesulfonamide (FOSA) on microplastics. Chemosphere. DOI: 10.1016/j.chemosphere.2014.08.047 | Water | PFOS and FOSA | Partitioning controlled by PFAS functional group | Compared PFOS and FOSA partitioning onto PE, PS, and PVC and demonstrated the importance of ionization state, hydrophobicity, pH, and salinity. |

**Abbreviations**: C4, C6, and C8, PFAS containing fluorinated carbon chains with four, six, and eight carbon atoms, respectively; DWR, durable water-repellent; F-53B, 6:2 chlorinated polyfluorinated ether sulfonate; FOSA, perfluorooctanesulfonamide; FTOH, fluorotelomer alcohol; FTSA, fluorotelomer sulfonic acid; 6:2 FTOH, 6:2 fluorotelomer alcohol; 6:2 FTSA, 6:2 fluorotelomer sulfonic acid; GenX, trade name commonly associated with hexafluoropropylene oxide dimer acid and its ammonium salt; HFPO-DA, hexafluoropropylene oxide dimer acid; MP, microplastic; NP, nanoplastic; PA, polyamide; PFAA, perfluoroalkyl acid; Pb, lead; PE, polyethylene; PET, polyethylene terephthalate; PFAS, per- and polyfluoroalkyl substances; PFBA, perfluorobutanoic acid; PFCA, perfluoroalkyl carboxylic acid; PFOA, perfluorooctanoic acid; PFOS, perfluorooctane sulfonate; PFSA, perfluoroalkyl sulfonic acid; PLA, polylactic acid; PP, polypropylene; PS, polystyrene; PVC, polyvinyl chloride; n:2 FTOH, fluorotelomer alcohol containing n fluorinated carbon atoms and two non-fluorinated carbon atoms.
